# Visual processing of manipulable objects in the ventral stream is modulated by parietal action systems

**DOI:** 10.1093/braincomms/fcag293

**Published:** 2026-07-27

**Authors:** Frank E Garcea, Emma Strawderman, William Burns, Matthew Cotroneo, Steven P Meyers, Tyler Schmidt, Kevin A Walter, Webster H Pilcher, Bradford Z Mahon

**Affiliations:** Department of Neuroscience, University of Rochester Medical Center, Rochester, NY 14642, USA; Department of Neurosurgery, University of Rochester Medical Center, Rochester, NY 14642, USA; Department of Brain & Cognitive Sciences, University of Rochester, Rochester, NY 14627, USA; Department of Neuroscience, University of Rochester Medical Center, Rochester, NY 14642, USA; Department of Neurosurgery, University of Rochester Medical Center, Rochester, NY 14642, USA; Department of Neurosurgery, University of Rochester Medical Center, Rochester, NY 14642, USA; Department of Neurosurgery, University of Rochester Medical Center, Rochester, NY 14642, USA; Department of Neurosurgery, University of Rochester Medical Center, Rochester, NY 14642, USA; Department of Brain & Cognitive Sciences, University of Rochester, Rochester, NY 14627, USA; Department of Imaging Sciences, University of Rochester Medical Center, Rochester, NY 14642, USA; Department of Neurosurgery, University of Rochester Medical Center, Rochester, NY 14642, USA; Department of Neurosurgery, University of Rochester Medical Center, Rochester, NY 14642, USA; Department of Neurosurgery, University of Rochester Medical Center, Rochester, NY 14642, USA; Department of Neurosurgery, University of Rochester Medical Center, Rochester, NY 14642, USA; Department of Psychology, Carnegie Mellon University, Pittsburgh, PA 15213, USA; Neuroscience Institute, Carnegie Mellon University, Pittsburgh, PA 15213, USA

**Keywords:** voxel-based lesion-activity mapping, manipulable objects, dorsal stream, connectome-based lesion-activity mapping, ventral stream

## Abstract

Functional object use requires the integration of visuomotor representations processed in the dorsal stream with representations of the visual form, surface texture and the material composition of objects, processed in the ventral stream. How do regions across the ventral and dorsal stream interact in support of functional object grasping and use? Here we show that the left inferior parietal lobe exerts a direct effect on neural responses in ventral occipitotemporal cortex (OTC) during visual processing of manipulable objects. We studied a series of consecutively enrolled participants in the pre-operative phase of their neurosurgical care (*N* = 109) with lesions distributed throughout the left hemisphere. Participants completed a category localizer functional MRI experiment in which they viewed images of manipulable objects, animals, faces and places. Using voxel-based lesion-activity mapping (VLAM), category preferences in neural responses in left ventral OTC were used to predict variance in voxelwise lesion incidence throughout the brain. This approach provides direct causal evidence about which regions outside of OTC, when lesioned, cause changes in neural responses within OTC. We found that lesions to the left anterior intraparietal sulcus and left supramarginal gyrus, two inferior parietal regions known to support object-directed grasping and manipulation, respectively, are associated with reduced neural responses for manipulable objects (compared to faces, places and animals) in the left ventral OTC. Parietal lesions do not modulate neural responses during visual processing of places in the same region of the left ventral OTC, even though places elicit stronger responses in the left ventral OTC than manipulable objects. To explore the structural pathways that may mediate these effects of diaschisis, we repeated the analysis using diffusion MRI-measured white matter fibre integrity as the dependent variable (instead of lesion location). That analysis identified the descending portion of the left arcuate fasciculus as the most likely pathway mediating the parietal-to-temporal lobe diaschisis identified in the VLAM analysis. These integrated VLAM and connectometry analyses demonstrate that inputs from parietal regions supporting skilled object-directed action shape neural responses in the ventral stream when viewing manipulable objects.

## Teaser

Garcea *et al*. show that lesions to parietal areas involved in object grasping and manipulation cause reduced neural responses in occipitotemporal regions when viewing manipulable objects. Diffusion tractography identified the descending portion of the arcuate fasciculus as a pathway mediating parietal-to-temporal lobe connectivity that supports functional object grasping and use.

## Graphical Abstract

**Graphical Abstract:**
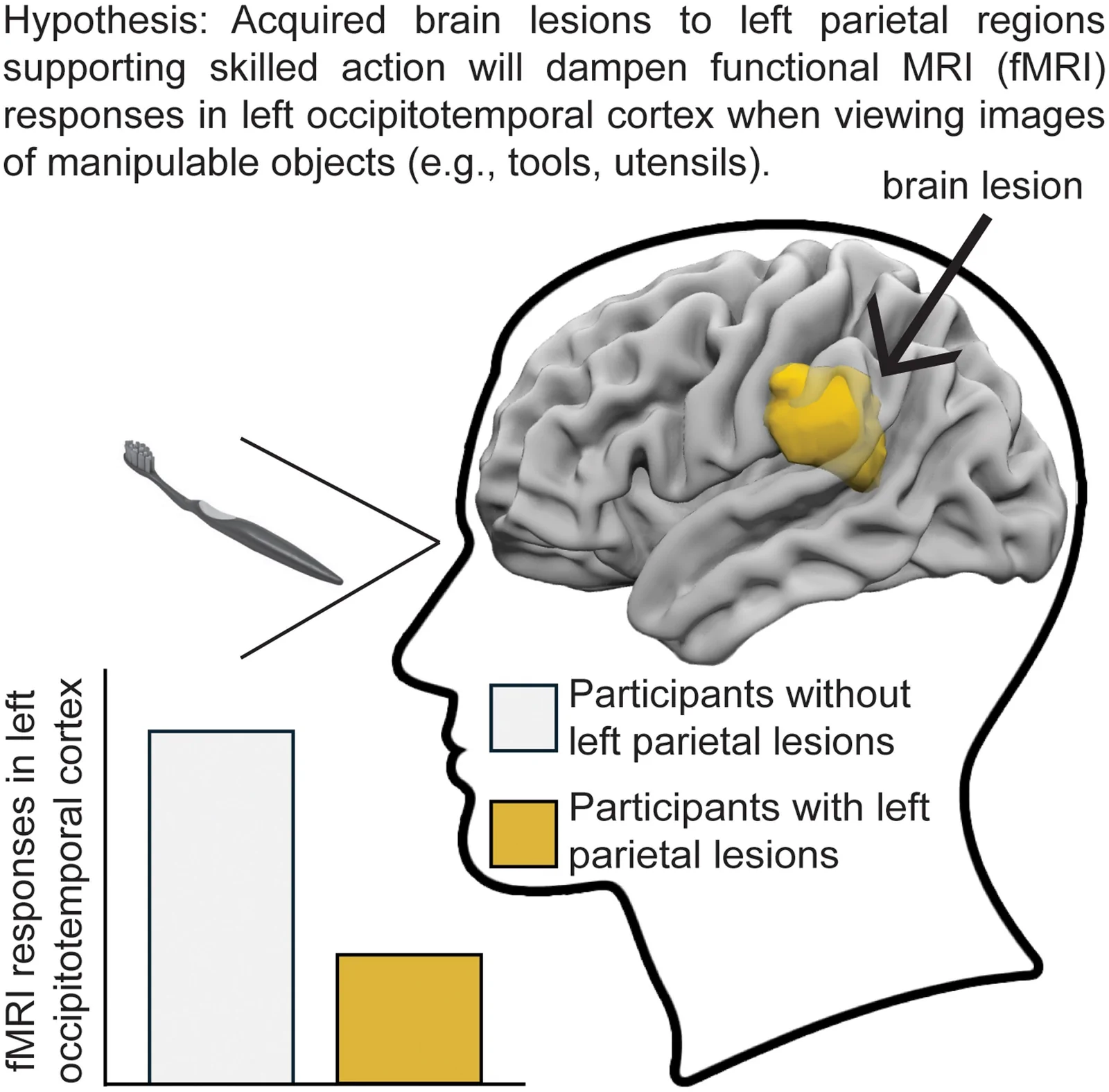

## Introduction

Every action performed upon an object—grasping a cup, opening a door and cutting an apple—requires coordinated processing across a network of temporal, parietal and frontal regions that support functional object grasping and use.^1-6^ The dorsal stream, which projects cortically and subcortically to posterior parietal regions, culminating in the anterior intraparietal sulcus (aIPS), processes visually based volumetric properties of objects and their spatial positions with respect to the body.^7-10^ Dorsal stream computations are sufficient to support biomechanically and volumetrically appropriate grasping. Grasping in a way that anticipates subsequent use (functional grasping) and skilled object manipulation (praxis) requires analysis of object properties such as shape, surface texture, material composition and function. Those object properties are computed by the ventral stream.^11^ For instance, the grip force required to grasp a slippery glass, or the hand posture to grasp an upside-down glass on a counter, depends on perceptual analysis that is supported by occipitotemporal regions^11-13^ (for discussion, see Mahon and Almeida^14^). If the action plan requires grasping a cup to take a drink, calibration of grip force will drive perceptual analysis of the material properties of the cup (e.g. is it Styrofoam or ceramic?) to determine if it might collapse under a certain grip force.^14-16^ The aIPS and supramarginal gyrus (SMG), of the inferior parietal lobule, support object-directed grasping and praxis, respectively.^9,17-22^ A key open issue is how dorsal and ventral pathways interact, and specifically whether processing in occipitotemporal regions is causally dependent on processing in parietal areas involved in object-directed grasping and use. A second open issue is which white matter pathways might support the integration of parietal-based action representations with occipitotemporal object representations.

Longitudinal fibres within the ventral stream, including the inferior longitudinal fasciculus and inferior fronto-occipital fasciculus, integrate processing along the hierarchy of visual regions—and support both feedforward and feedback processing.^23-25^ Traditionally, models of visual object recognition have emphasized that visual perceptual analysis is driven by processes internal to the ventral stream.^8,26-29^ The ventral stream is defined computationally as a pathway that supports visual object identification. Without challenging the maxim that the ventral stream supports identification, there are multiple ways in which computations specific to an object’s identity are used in the service of behaviour, beyond identification or ‘labelling’. The outputs of ventral stream processing interface with somatosensory inputs, memory, language, action and many other systems. Here, we focus specifically on the interaction of ventral occipitotemporal cortex (OTC) with parietal and frontal regions that calibrate object-directed actions.^6,22,30,31^ We test the hypothesis that parietal-based action computations provide inputs to occipitotemporal processes supporting the visual analysis of manipulable objects.

Visual processing of manipulable objects drives differential blood oxygen level-dependent (BOLD) responses in specific regions along ventral OTC, in particular along the collateral sulcus and the medial fusiform gyrus.^32,33^ Those regions are functionally defined as exhibiting stronger BOLD responses to manipulable objects compared to faces, animals and written words.^32,34-41^ Images of places and highly contextualized objects (i.e. landmarks, large non-manipulable objects) differentially drive BOLD responses in the parahippocampal gyrus,^42,43^ which is anterior to ventral OTC voxels exhibiting preferences for manipulable objects.^44,45^ However, even within the collateral sulcus and medial fusiform area that exhibits stronger responses to manipulable objects than faces or animals, places and large non-manipulable objects typically elicit stronger BOLD responses than manipulable objects.^15,33,45^

Interestingly, other functional MRI (fMRI) indices point to neural specificity for manipulable objects in the collateral sulcus and medial fusiform gyrus. For instance, the voxelwise pattern of preferences for (specifically) manipulable objects in left ventral OTC is related to the voxelwise pattern of functional connectivity to (specifically) the left SMG.^44^ More generally, the ventral OTC regions exhibiting neural preferences for manipulable objects also exhibit differential functional connectivity to the left aIPS and SMG.^1,46-48^ Focal lesions along the collateral sulcus cause deficits for knowledge of object surface texture and material properties^49,50^ (for convergent fMRI findings, see Cant and Goodale^51^ and Cant *et al*.^52^), and there is some indication that lesions to those structures can differentially affect processing of manipulable objects.^53^ Thus, on the one hand, the amplitude of BOLD responses shows one pattern (stronger for places than manipulable objects^45^), while patterns of repetition suppression,^33^ functional connectivity^44^ and lesion evidence^49^ demonstrate various degrees of specialization for processing *manipulable* objects in the collateral sulcus and medial fusiform gyrus.

By hypothesis, fMRI responses in left ventral OTC when viewing images of manipulable objects represent a complex summation of processing across both the ventral and dorsal streams. Some of the inputs to ventral OTC come from action-relevant processing in the parietal cortex.^33,40^ We refer to this framework as a dorsal-to-ventral model. Here, we seek evidence for the dorsal-to-ventral model by testing (i) if lesions to parietal regions disrupt fMRI responses in left ventral OTC for manipulable objects and (ii) by identifying which white matter pathways mediate the effect of parietal lobe lesions on distal fMRI responses in left ventral OTC.

We analysed data from 109 individuals with acquired brain lesions and 55 neurotypical participants. Each participant completed an fMRI task involving manipulable objects, animals, faces and places, as well as a diffusion-weighted MRI. An issue for traditional voxel-based lesion-symptom mapping studies is to ensure that clinical variables across the patient sample, which can affect the fMRI signal (e.g. oedema, speed of tumour growth), are not confounds in relating lesion location to performance measures. Voxel-based lesion-activity mapping (VLAM) offers an innovative way to manage this concern, as it is based on relating lesion location to variance in a within-subject contrast computed from an fMRI study. In the present case, we test the effect of brain lesions in the parietal cortex on BOLD responses for visual processing of manipulable object and place stimuli in left ventral OTC. In this way, the test of specificity to manipulable objects, compared to places, is within-participant and thus orthogonal to between-participant variance in clinical factors.

We first show that left SMG and aIPS lesions are associated with reduced BOLD responses in left ventral OTC during visual processing of manipulable objects—but not during processing of place stimuli, extending a prior study in a smaller group of participants.^15^ We then use connectometry to describe the white matter pathways that mediate structural connectivity between the identified parietal regions and left ventral OTC. Candidate tracts that integrate object representations in OTC with skilled action production systems in left parietal cortex include the arcuate fasciculus,^54^ the parietal aslant tract,^55^ the vertical occipital fasciculus,^56,57^ the middle longitudinal fasciculus^58^ and the inferior fronto-occipital fasciculus.^59^ We find that reduced structural integrity of the descending portion of the left arcuate fasciculus is associated with reduced BOLD responses to manipulable objects, but not for places, in left ventral OTC. This indicates a role for the descending arcuate fasciculus in supporting functional object-directed action.

## Materials and methods

### Participants

One hundred and fourteen individuals with an acquired brain lesion participated in the study. Participants had normal or corrected-to-normal vision and were in the pre-operative phase of their neurosurgical care (49 females; mean age, 46.54 years; SD 16.48; see Supplementary Table 1 for demographic variables and Supplementary Fig. 1 for lesion overlap among participants). Participants took part in a T1 anatomical scan, a visual category localizer fMRI experiment, diffusion-weighted imaging, and resting state fMRI (data not analysed herein). Fifty-five neurotypical adults participated in the same MRI protocol as part of a separate project investigating object representations in the ventral stream (29 females; mean age, 22.27 years; SD 6.03 years).^60-64^ Neurotypical participants had no history of psychiatric illness or neurologic injury and were right-hand dominant (established with the Edinburgh handedness questionnaire^65^). All participants had normal or corrected-to-normal vision, took part in the study in exchange for payment and gave written informed consent in accordance with the University of Rochester Research Subjects Review Board and an sIRB with Carnegie Mellon University.

### Visual category localizer functional MRI experiment

fMRI data collection occurred over an 8-year period. Stimulus presentation was controlled with ‘A Simple Framework’^66^ using the Psychophysics Toolbox,^67^ PsychoPy^68^ or with custom stimulus presentation software. Data for 5 of the 114 participants with lesions were removed due to technical issues with stimulus presentation software. Participants viewed the stimuli binocularly through a mirror attached to the head coil adjusted to allow foveal viewing of a back-projected monitor (spatial resolution = 1400 × 1050 pixels; temporal resolution = 120 Hz). Each MRI scan consisted of two to eight runs of the category localizer experiment. Several updates were introduced over the years to the category localizer experiment, resulting in three versions. Details on how the category localizer paradigm differed, and MRI data acquisition parameters, are provided in the Supplementary material. Of primary relevance here is what was common across all versions of the paradigm: Images of manipulable objects, faces, animals and places presented within a mini-block design, blocked by category, within a silent naming task.

### Lesion identification

Lesions were delineated on each participant’s native T1-weighted anatomical image by separating pathological from intact tissue. Voxels containing damaged grey or white matter were coded with a value of 1, whereas preserved tissue voxels were assigned a value of 0 using ITK-SNAP. All participants provided written informed consent permitting the research team to access clinical MRI scans obtained during routine clinical care. Lesion masks were reviewed by a neuroradiologist (co-author S.P.M.), who was blinded to the study hypotheses at the time of review and had access to T1-weighted images, T2-weighted images and FLAIR scans with and without contrast. Lesion reconstructions were visually inspected and, when necessary, refined by members of the study team to ensure accurate delineation of lesion boundaries.

Lesion masks defined in native T1 space were incorporated as cost-function masks^69^ during anatomical and functional pre-processing. This procedure reduces normalization errors associated with lesioned tissue while preserving accurate registration of anatomical data into Montreal Neurological Institute (MNI) space. Corrected lesion masks were subsequently transformed into MNI space using the same ANTs-derived transformation matrix applied to normalize the native T1 anatomical image. Following normalization, lesion maps in standard space were visually reviewed and verified against anatomical landmarks to confirm that no distortions had been introduced during spatial transformation.^15,70^

### Anatomical MRI pre-processing

For each participant, T1-weighted anatomical images were corrected for intensity non-uniformity using ANTs’ N4BiasFieldCorrection and subsequently used as the T1 reference image. Skull stripping of the T1 reference was performed with the antsBrainExtraction.sh pipeline implemented in ANTs, using the OASIS30ANTs template as the extraction target. Tissue segmentation of grey matter, white matter and cerebrospinal fluid was conducted on the brain-extracted T1 image using FSL’s fast algorithm. Spatial normalization to MNI152NLin2009cAsym standard space was achieved through non-linear registration with antsRegistration, using brain-extracted versions of both the participant T1 image and template. The ICBM 152 Nonlinear Asymmetrical 2009c template served as the normalization target.

### Functional MRI pre-processing

For each functional run, fMRIPrep generated a reference BOLD volume along with a skull-stripped version of that reference image.^71^ Head-motion estimates relative to the BOLD reference image, including rigid-body transformation matrices and six motion parameters describing translation and rotation, were calculated prior to spatiotemporal filtering (‘mcflirt’, FSL). Slice-timing correction was applied to each BOLD run with AFNI’s 3dTshift. Following motion correction, BOLD time series were resampled back into native space by applying the estimated transforms. The BOLD reference image was co-registered to the participant’s T1 reference (‘mri_coreg’, FreeSurfer) followed by boundary-based registration implemented (‘flirt’, FSL). Registration was performed with twelve degrees of freedom to accommodate residual distortions present in the functional reference image. Functional data were subsequently normalized into MNI152NLin2009cAsym space, yielding pre-processed BOLD runs in MNI space.

After pre-processing, anatomical and functional images were analysed using BrainVoyager together with in-house MATLAB scripts incorporating the NeuroElf toolbox. Functional volumes were interpolated to 3 × 3 × 3 mm resolution and underwent spatial smoothing with a 6 mm FWHM Gaussian kernel as well as temporal high-pass filtering (cut-off: 2 cycles per run). For each participant, experimental effects were estimated with a general linear model in which task events were convolved with a canonical two-gamma haemodynamic response function. First-derivative motion parameters from 3D motion correction were included as nuisance regressors to account for variance associated with participant movement.

### Diffusion MRI pre-processing

FSL’s BET tool was used to skull-strip each participant’s diffusion and T1 images, as well as the fieldmap magnitude image. The B0 image was stripped from the diffusion-weighted image, and the fieldmap was prepared using FSL’s prepare fieldmap tool. Smoothing and regularization were performed using FSL’s fugue tool and a 3D Gaussian smoothing was applied (sigma = 4 mm). The magnitude image was warped based on this smoothing, with *y* as the warp direction. Eddy current correction was performed (‘eddy_correct’, FSL), which takes each volume of the diffusion-weighted image and registers it to the b0 image to correct for both eddy currents and motion. The deformed magnitude image was registered to the b0 image (‘flirt’, FSL). The resulting transformation matrix was then applied to the prepared fieldmap. The diffusion-weighted image was undistorted using the registered fieldmap (‘fugue’, FSL). Intensity correction was also applied to this unwarping. This procedure was applied to all but three participants. Susceptibility artefact in the three remaining datasets was corrected using FSL’s TOPUP tool. The outputs of TOPUP served as inputs to FSL’s eddy correct tool.

### Functional definition of manipulable object preferring voxels in left ventral occipitotemporal cortex

VLAM uses fMRI responses in a reference ROI to predict lesion overlap throughout the brain. Thus, the first step in the analysis is to identify, for each participant, the specific voxels in left ventral OTC that exhibit the strongest preferences for manipulable objects, compared to baseline categories. In the second step of the analysis, those participant-specific values of the peak ‘category effect’ for manipulable objects serve as the independent variable in a whole-brain analysis that predicts lesion likelihood.

Two approaches were taken to defining the peak of manipulable object preferences in left ventral OTC. The first analysis sought to optimize the definition of the peak for the contrast of ‘Tools > [Animals, Faces, and Places]’ (equally weighted), in each participant. The second analysis sought to optimize the independence of voxel definition and extraction of peak values from those voxels. We note that, for both approaches, the resulting measure of manipulable object preferences in left ventral OTC serves as the independent variable in a subsequent whole-brain VLAM analysis (see below). Thus, regardless of how manipulable object preferring voxels in left ventral OTC are functionally defined in the first step of the analysis, the VLAM analysis avoids ‘double dipping’^72^ and is not circular, as it is directed at an entirely new dependent measure (lesion location). Unless otherwise noted, analyses of VLAM effects reported below are based on the first approach to defining manipulable object preferences described next.

**Figure 1:**
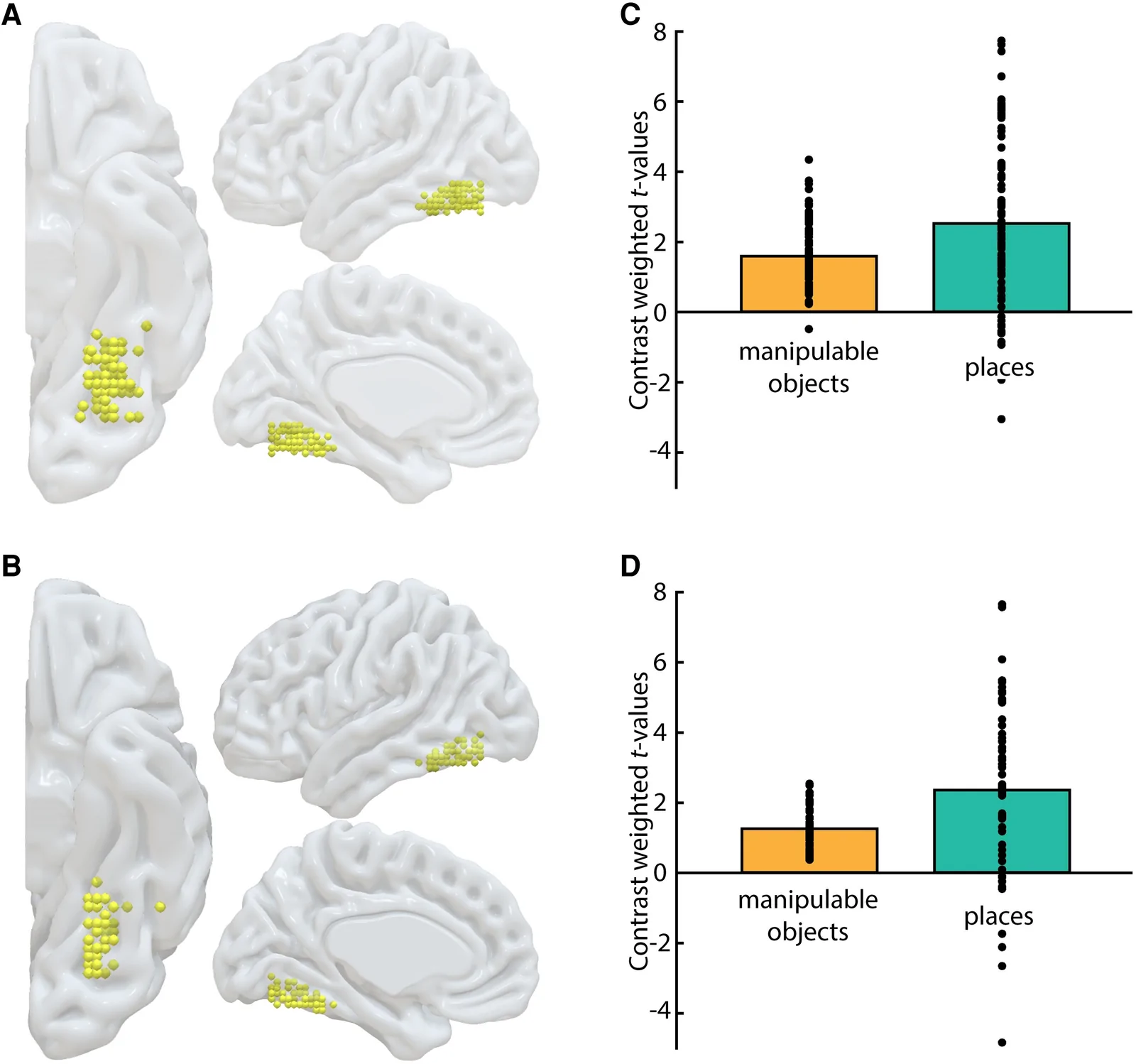
**Preferences for manipulable objects and places in the left ventral OTC.** Each data point represents the participant-specific centroids exhibiting preferences for manipulable objects in the left ventral OTC in 95 participants with lesions (**A**) and 52 neurotypical participants (**B**). A leave-one-out cross-validation approach used a spherical ROI localized using *N*-1 runs of data to extract manipulable object preferences from the *N*th run using the contrast of ‘Tools > [Animals, Faces and Places]’ (equally weighted). This procedure was iterated *N* times and averaged across *N* data folds to derive a cross-validated *t*-value for manipulable objects. The same ROIs were used to obtain cross-validated *t*-values for place stimuli using the contrast of ‘Places > [Animals, Faces and Tools]’ (equally weighted). Each dot is a data point representing the participant-specific cross-validated response for manipulable objects and place stimuli in participants with lesions (**C**) and neurotypical participants (**D**). Using a mixed ANOVA, we found a main effect of Category (*F*(1, 145) = 20.56, *P* < 0.001, *ƞ*^2^ = 0.12), as place responses were stronger than manipulable object responses. There was no main effect of Group, nor an interaction between Group and Category (*F* < 1), indicating equivalently larger fMRI responses for places than manipulable objects in participants with lesions compared to neurotypical participants.

In the first approach for defining manipulable object preferences in left ventral OTC, the whole-brain contrast of ‘Tools > [Animals, Faces and Places]’ (equally weighted) was computed at the participant level (using all runs of data). If this contrast did not elicit a focus in left ventral OTC below a false discovery rate (FDR) corrected value (*q* < 0.05), the threshold was relaxed to *P* < 0.05, uncorrected. This localization scheme identified voxels preferring manipulable objects in left ventral OTC in 95 of the 109 participants with brain lesions (88%; Fig. 1A) and 52 of the 55 neurotypical participants (96%; Fig. 1B; see Supplementary Table 1). A spherical ROI 6 mm in diameter was drawn on the peak (see Fig. 1A). An iterative process was then carried out within that ROI, using *N*-1 runs to find the peak voxel and the *N*th run to extract the contrast-weighted *t*-value for ‘Tools > [Animals, Faces and Places]’ (equally weighted). At each iteration, OTC voxels that preferred manipulable objects defined using *N*-1 runs were also used to extract the contrast-weighted *t*-value for the contrast of ‘Places > [Animals, Faces and Tools]’ (equally weighted). Averaging over folds resulted in a mean contrast-weighted *t*-value for manipulable object preferences and place preferences, for each participant, for manipulable object preferring voxels in left ventral OTC (see Fig. 1C and D).

In the second approach, the whole-brain contrast of ‘Tools > [Animals, Faces and Places]’ (equally weighted) was computed over even runs, and separately, over odd runs, at the participant level. If this contrast did not elicit a response below an FDR-corrected value (*q* < 0.05), the threshold was relaxed to *P* < 0.05, uncorrected. This localization scheme identified voxels preferring manipulable objects in left ventral OTC in 64 of the 109 participants with brain lesions (see Supplementary Table 1). A spherical ROI 6 mm in diameter was drawn on the peak voxel in MNI space. The ROI from even runs was used to extract the *t*-values from contrast of ‘Tools > [Animals, Faces and Places]’ (equally weighted) from odd runs, and vice versa. Averaging over folds resulted in mean contrast-weighted *t*-values for manipulable object preferences and place preferences.

### Statistical analysis 1: voxel-based lesion-activity mapping

VLAM was performed with the SVR-LSM toolbox using a univariate model.^73^ Prior to the VLAM analysis, each participant’s lesion mask was interpolated from 1 × 1 × 1 to 2 × 2 × 2 mm to reduce the number of multiple comparisons performed. Voxels lesioned in at least 10% of participants were analysed. We controlled for variability in lesion volume using the ‘Regress Both’ function, which regresses variability in total lesion volume from fMRI responses and from every lesioned voxel entered in the analysis. Voxelwise statistical significance was determined using a permutation analysis in which alignment of the independent variable (fMRI responses) and the dependent variable (lesion maps) was randomly shuffled across participants, repeating the full analysis 10 000 times. Voxelwise *Z*-scores were computed for the true data in relation to the mean and standard deviation of voxel-by-voxel null distributions; the resulting *Z*-score map was set to a threshold of *z* < −1.65 (*P* < 0.05, one-tailed) to determine chance-level likelihood of identifying a significant effect in each voxel. If the resulting voxelwise clusters did not survive permutation analysis, we removed clusters with fewer than 500 contiguous voxels before interpreting the results.^74-78^ The analysis was then re-run using place preferences extracted from the participant-specific manipulable object preferring ROIs. The Harvard-Oxford cortical and subcortical probabilistic atlases, as well as parietal regions from the Julich Brain atlas, assessed the location of significant voxels identified using VLAM.

### Statistical analysis 2: connectome-based lesion-activity mapping

Seventy-five of the 95 participants in whom we could localize manipulable object-preferring voxels in left ventral OTC took part in the 60-direction diffusion MRI protocol and were thus included in the white matter fibre tracking analysis. Of the 20 remaining participants, 1 did not take part in diffusion MRI and 19 took part in a 258-direction diffusion spectrum imaging protocol (data not analysed herein). Fractional anisotropy (FA) along each voxel of the eddy corrected diffusion MRI data was modelled within DSI Studio. Each participant’s FA map was co-registered to the ICBM152 template and entered into a connectome-wide analysis. The connectome-wide analysis uses whole-brain multiple regression with Spearman correlation to relate variance in voxelwise FA to variance in manipulable object preferences in left ventral OTC, after controlling for total lesion volume. Voxels in which FA was positively associated with the strength of manipulable object preferences in left ventral OTC were identified if the voxelwise correlation exceeded a *t*-statistic of 3.00. Next, the VLAM lesion site was entered as an ROI to ensure that identified white matter voxels meet the criterion of intersecting a tract that is structurally connected to the VLAM-identified sites. Finally, a 10 000-iteration permutation analysis was performed by randomly assigning fMRI responses to FA maps. For each permutation, multiple regression was performed and significant voxels were entered into an identical analysis to estimate the likelihood of identifying white matter tracts structurally connected to the VLAM-identified lesion site when randomly permuting the data. The length of fibre tracts identified in the permutation analysis was compared against the length of fibre tracts in the non-permuted analysis to estimate the FDR (*q* < 0.05), using a length threshold of 15. That connectometry analysis was then re-run using place preferences derived from participant-specific left ventral OTC ROIs.

## Results

### Manipulable object and place representations in left ventral occipitotemporal cortex

We tested if preferences for manipulable objects and places in left ventral OTC were comparable between the lesion and neurotypical participant groups, using a mixed ANOVA (between-subject factor Group, within-subject factor Category). There was a main effect of Category (*F*(1, 145) = 20.56, *P* < 0.001, *ƞ*^2^ = 0.12), as place responses were stronger than manipulable object responses. This finding replicates prior studies^15,44,45^ and shows that non-manipulable stimuli (e.g. places, scenes, houses) drive, if anything, stronger fMRI responses than manipulable objects in left ventral OTC voxels. There was no main effect of Group, nor an interaction between Group and Category (*F* < 1), indicating equivalently larger fMRI responses for places than manipulable objects in participants with lesions (Fig. 1C) compared to neurotypical participants (Fig. 1D). This observation establishes ‘places’ as a stringent baseline against which to test whether parietal lesions disrupt neural responses for visual processing of, specifically, manipulable objects in the left ventral OTC.

### Supramarginal gyrus and anterior intraparietal sulcus lesions modulate blood oxygen level-dependent responses for visual processing of manipulable objects in left ventral occipitotemporal cortex

**Figure 2:**
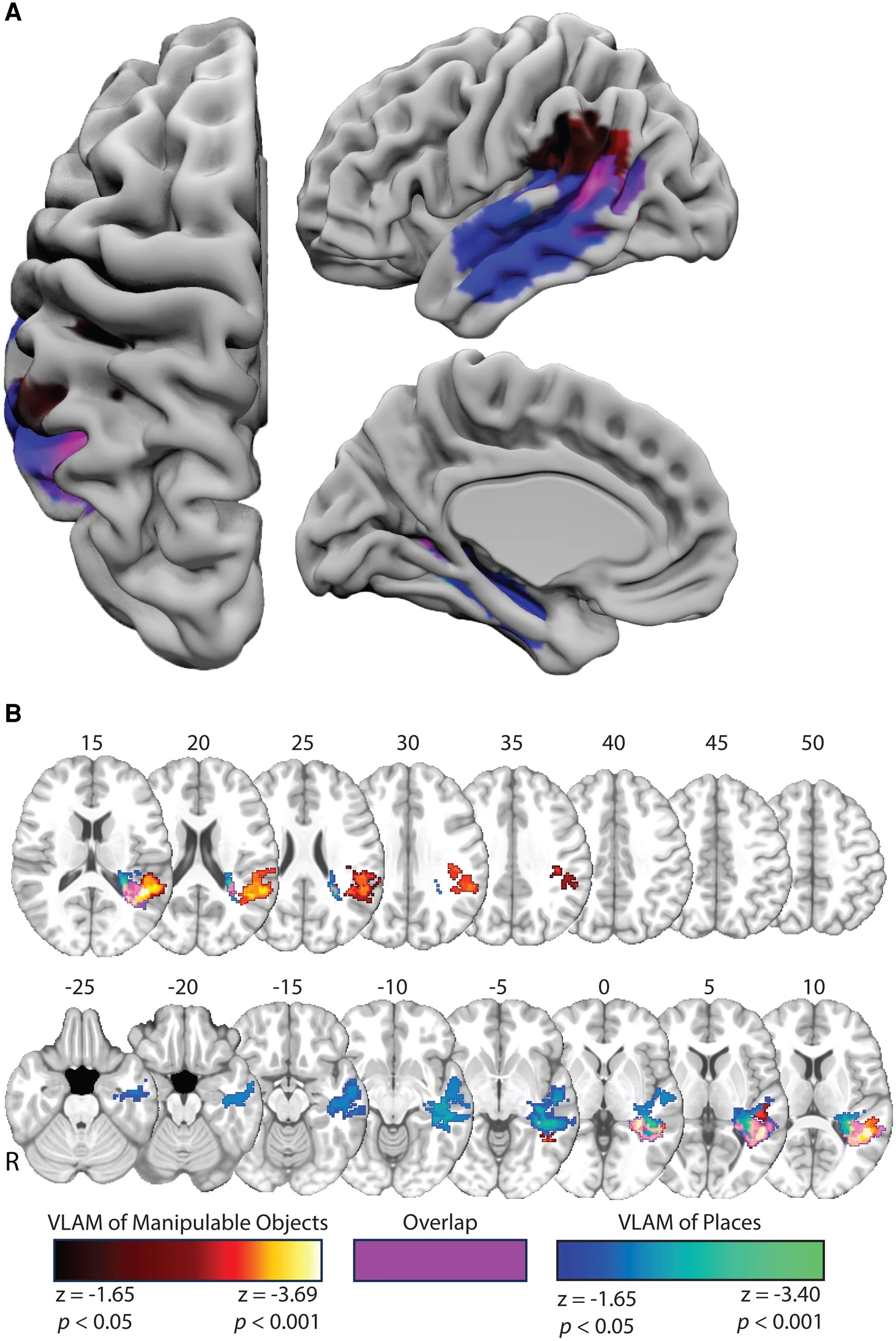
**VLAM analysis of manipulable objects and places in the left ventral OTC.** (**A**) VLAM is a statistical technique that relates the strength of fMRI responses in a given ROI to the presence or absence of lesion damage in any given voxel of the brain. Voxelwise statistical significance was determined using a permutation analysis in which alignment of the independent variable (fMRI responses), and the dependent variable (lesion maps) was randomly shuffled across participants, repeating the full analysis 10 000 times. Voxelwise *Z*-scores were computed for the true data in relation to the mean and standard deviation of voxel-by-voxel null distributions; the resulting *Z*-score map was set to a threshold of *z* < −1.65 (*P* < 0.05, one-tailed) to determine chance-level likelihood of identifying a significant effect in each voxel. Each voxel is thus a single data point in the analysis. In a VLAM analysis with 95 participants, we find that reduced fMRI responses for manipulable objects in left ventral OTC were associated with lesion presence in the left SMG, aIPS, parietal operculum, angular gyrus and the posterior middle temporal gyrus. An overlapping lesion site in the posterior temporal lobe was also associated with reduced place preferences, whereas lesions to anterior portions of the temporal lobe, thalamus, insula, hippocampus and posterior fusiform gyrus were associated specifically with reduced fMRI responses for visual processing of places in left ventral OTC. (**B**) A volumetric rendering is provided with MNI Z coordinates listed for each axial image.

**Figure 3:**
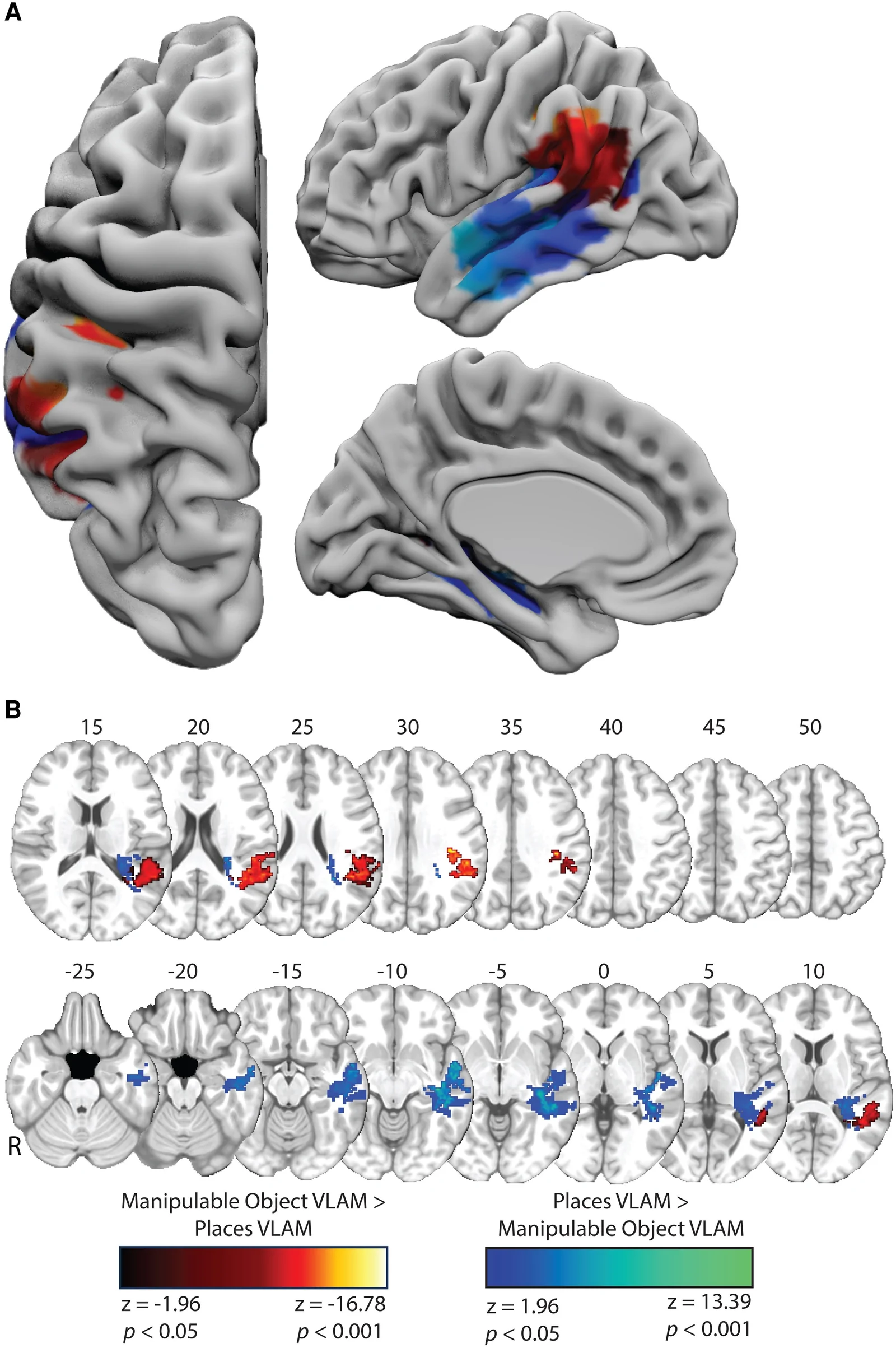
**Voxelwise comparison between VLAM analyses of manipulable objects and places in the left ventral OTC.** With the maps obtained from the VLAM analyses with 95 participants (Fig. 2), we performed an analysis in each voxel to quantify the difference between the VLAM result for manipulable objects and place stimuli. A 1000-iteration permutation analysis followed, in which the VLAM maps were scrambled voxelwise prior to subtracting the place VLAM map from the manipulable object VLAM map. The voxelwise difference scores of the true data were then *Z*-scored against the mean permutation-derived voxelwise difference scores. Thus, each voxel is a single data point in the analysis. (**A**) The voxelwise comparison identifies the left SMG, aIPS, posterior middle temporal gyrus and underlying white matter as a VLAM-identified lesion site that was significantly greater for manipulable objects than places. By contrast, lesions to the anterior inferior, middle and superior temporal gyri, as well as the thalamus, insula, hippocampus and posterior fusiform gyrus, were associated with a stronger VLAM result for places. (**B**) A volumetric rendering is provided with MNI Z coordinates listed for each axial image.

In the core VLAM analysis, we used the contrast-weighted *t*-values for manipulable object preferences in the left ventral OTC as the independent variable (Fig. 1B) to predict lesion likelihood throughout the brain (dependent variable). That analysis showed that left inferior parietal lobule (LIPL) lesions were associated with lower amplitude BOLD responses in left ventral OTC for visual processing of manipulable objects. This included the SMG, extending into the aIPS, parietal operculum and angular gyrus (Table 1; see Supplementary Table 2 and Figs 2 and 3). The analysis also identified the posterior middle temporal gyrus as a lesion site associated with reduced BOLD responses for manipulable objects in left ventral OTC (Fig. 2A, red). This VLAM result extends a prior demonstration in 35 participants with lesions.^15^

**Table 1:** Left hemisphere locations of highest lesion concentration, identified in the VLAM analyses using left ventral OTC neural preferences for manipulable objects and places

Analysis	Region name	Peak MNI coordinate (XYZ)			Statistical value	Number of significant voxels
Manipulable objects	Posterior supramarginal gyrus	−47	−47	15	−3.09	234 (20.2%)
Manipulable objects	Middle temporal gyrus, temporal-parietal-occipital territory	−48	−46	6	−2.93	91 (10.0%)
Manipulable objects	Angular gyrus	−48	−51	20	−2.62	100 (9.0%)
Manipulable objects	Hippocampus	−30	−38	0	−2.60	19 (2.6%)
Manipulable objects	Planum temporale	−49	−40	20	−2.32	99 (17.1%)
Manipulable objects	Parietal operculum	−50	−32	20	−2.28	156 (26.6%)
Manipulable objects	Posterior superior temporal gyrus	−57	−38	8	−2.08	9 (2.1%)
Manipulable objects	Anterior supramarginal gyrus	−65	−28	20	−1.72	16 (1.7%)
Places	Posterior middle temporal gyrus	−50	−38	−2	−2.71	326 (22.3%)
Places	Middle temporal gyrus, temporal-parietal-occipital territory	−52	−50	6	−2.69	69 (7.6%)
Places	Hippocampus	−26	−40	1	−2.43	62 (8.6%)
Places	Posterior superior temporal gyrus	−62	−18	−2	−2.43	13 (3.1%)
Places	Planum temporale	−42	−30	7	−2.32	29 (5.0%)
Places	Planum polare	−44	−14	−2	−2.11	39 (10.3%)
Places	Heschl’s gyrus	−46	−26	8	−2.03	18 (5.1%)
Places	Posterior fusiform gyrus	−38	−18	−22	−1.9	30 (3.3%)
Places	Anterior superior temporal gyrus	−51	−8	−14	−1.87	77 (25.0%)
Places	Anterior middle temporal gyrus	−52	−6	−24	−1.87	67 (13.8%)
Places	Posterior supramarginal gyrus	−47	−47	15	−1.82	17 (1.5%)

The number of significant voxels in each region from the Harvard-Oxford cortical and subcortical parcellation atlases is provided, and the percentage of significant voxels in each atlas region is listed in parentheses (minimum threshold of 1%).

We then re-ran the VLAM analysis using manipulable object preferences in left ventral OTC, defined using the even/odd approach for voxel definition and testing. Once again, we found that left SMG and aIPS lesions were associated with weaker responses to manipulable objects in left ventral OTC (see Supplementary Fig. 4). This effect establishes that the core VLAM finding is independent of how manipulable object preferences in ventral OTC are defined.

### Left inferior parietal lobule lesions modulate blood oxygen level-dependent responses specifically for manipulable objects, and not places, in left ventral occipitotemporal cortex

As noted in the Introduction, an important consideration for traditional voxel-based lesion-symptom mapping methods is that clinical variables that differ across participants may be correlated with the independent measure of interest. Note that all VLAM analyses herein statistically control for lesion size, which is a key clinical variable that can differ across participants. We also confirmed that differences in lesion aetiology do not account for the core findings (see Supplementary Fig. 5). However, the strongest demonstration that the core finding cannot be explained by confounding clinical variables would be to show that LIPL lesions do not affect BOLD responses for visual processing of place stimuli in left ventral OTC, in the same participants. This is because all clinical and demographic variables are held constant when comparing to another condition within the same fMRI study that participants completed.

When we repeated the VLAM analysis with place preferences as the independent variable, we identified a lesion site in the lateral temporal cortex, including the middle temporal and superior temporal gyri, and a portion of white matter undercutting the parietal operculum (Fig. 2, blue). A second site included the thalamus, insula and hippocampus (Table 1). Critically, the voxels identified in the VLAM analysis of places did not overlap with the parietal sites identified in the analysis of manipulable objects. The overlap between the two analyses was principally in the superior and middle temporal gyri, as well as in the left angular gyrus, areas associated with semantic representation and processing^79-81^ (Fig. 2, purple).

We then tested whether the VLAM results for manipulable objects were statistically different from the corresponding VLAM result for places, using a permutation test to estimate the likelihood of the observed differences. Figure 3 (red-to-yellow) shows voxels for which lesion presence *differentially* reduced BOLD responses to manipulable objects, compared to places, in left ventral OTC. This analysis again identifies the left SMG and aIPS regions, as identified in the original VLAM analysis of manipulable objects (Fig. 2). Smaller clusters of voxels were identified in the left parietal operculum and the left posterior middle temporal gyrus (Supplementary Table 3A). These results demonstrate that LIPL lesions—including cortical and subcortical structures—affect BOLD responses specifically for manipulable objects in left ventral OTC. This result is obtained using a stringent baseline—place stimuli, which elicit, if anything, stronger responses in left ventral OTC compared to manipulable objects (Fig. 1B).^44,45^ Figure 3 (blue-to-green) shows that lesions to the anterior inferior, middle and superior temporal gyri, the thalamus, insula and hippocampus differentially affected BOLD responses for places in left ventral OTC (Supplementary Table 3B). This extends a previous demonstration that lesions to these temporal lobe sites were associated with reduced responses for places in left ventral OTC^15^ (for discussion of temporal-parietal connectivity in support of visuospatial processing and navigation, see Kravitz *et al*.^82^).

### Connectome-based lesion-activity mapping of manipulable object and place preferences in ventral occipitotemporal cortex

Connectometry is a technique in which variance in FA throughout the brain is predicted by an independent variable,^83^ in this case, contrast-weighted *t*-values for manipulable object and place preferences in left ventral OTC (for other variants of this method, see Yourganov *et al*.^84^ and Gleichgerrcht *et al*.^85^). Variability in total lesion volume was regressed from the independent variable prior to conducting the analysis.

We first identified, whole-brain, voxels for which variance across participants in FA was related to variance in BOLD responses for visual processing of manipulable objects in left ventral OTC. We imposed the additional constraint that voxels had to be structurally connected to the VLAM-identified site for manipulable objects (Fig. 3, red-to-yellow). Thus, the voxels identified meet the criteria of (i) exhibiting a significant relation of variance in FA to BOLD responses to manipulable objects in left ventral OTC and (ii) are part of tracts that are structurally connected to the parietal voxels that exhibited a statistically stronger VLAM effect for manipulable objects compared to place stimuli and (iii) which are significantly longer (based on permutation testing) than 15 voxels. That entire analysis was then repeated, using place preferences in left ventral OTC as the independent variable. As with manipulable objects, the VLAM-identified lesion site for places (Fig. 3, blue-to-green) was used as an ROI to constrain the analysis to structurally connected white matter voxels.

**Figure 4:**
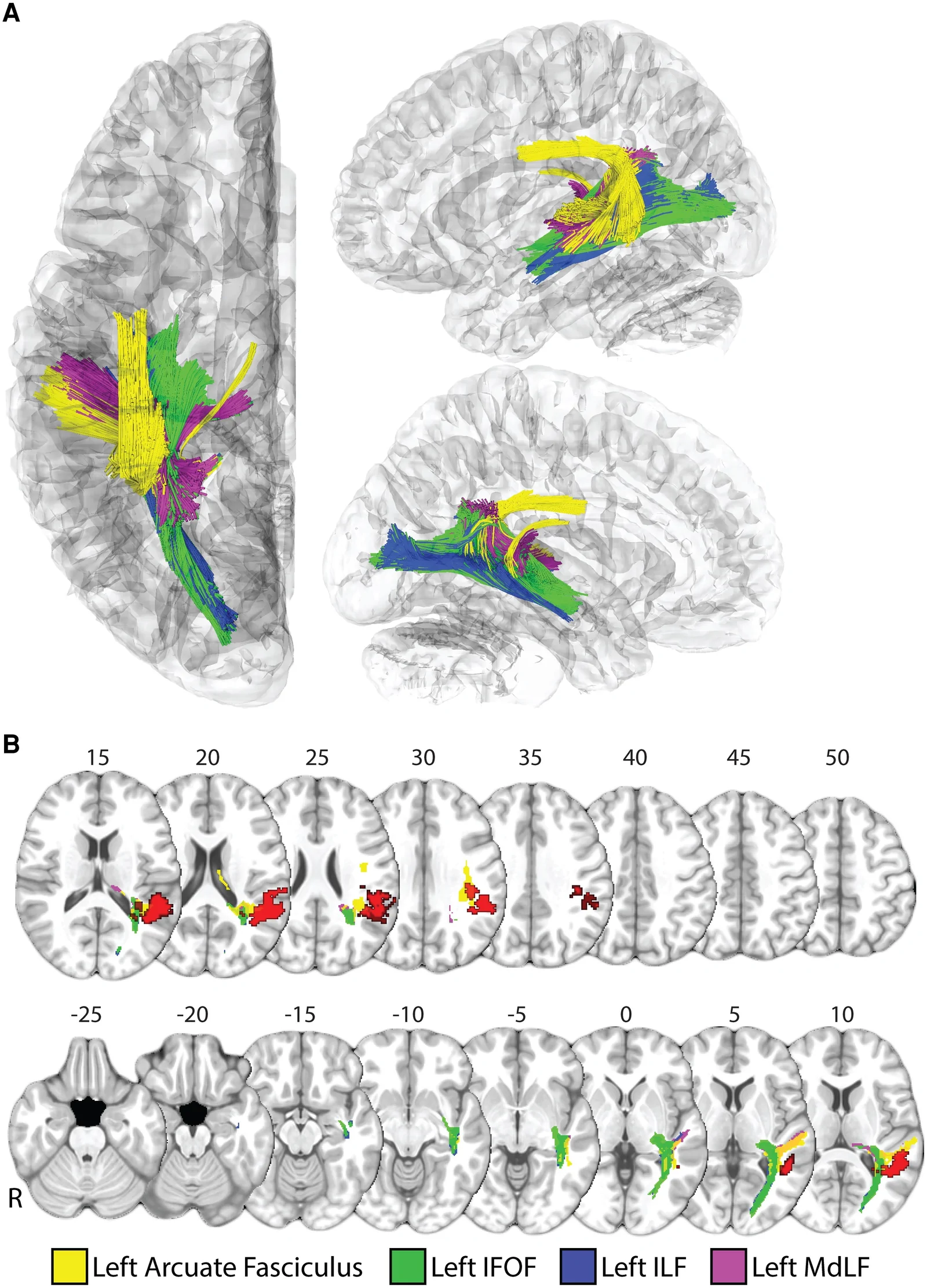
**Connectome-based lesion-activity mapping of manipulable objects in the left ventral OTC.** The connectome-wide analysis uses whole-brain multiple regression with Spearman correlation to relate variance in voxelwise FA to variance in manipulable object preferences in left ventral OTC, after controlling for total lesion volume. Voxels in which FA was positively associated with the strength of manipulable object preferences in left ventral OTC were identified if the voxelwise correlation exceeded a *t*-statistic of 3.00. Next, the VLAM lesion site (see Fig. 3, red-to-yellow) was entered as an ROI to ensure that identified white matter voxels meet the criterion of intersecting a tract that is structurally connected to the VLAM-identified sites. Finally, a 10 000 iteration permutation analysis was performed by randomly assigning fMRI responses to FA maps. For each permutation, multiple regression was performed and significant voxels were entered into an identical analysis to estimate the likelihood of identifying white matter tracts structurally connected to the VLAM-identified lesion site when randomly permuting the data. The resulting fibres represent a single data point in the analysis. This analysis was performed in 75 participants. (**A**) The left inferior fronto-occipital fasciculus (IFOF), the left inferior longitudinal fasciculus (ILF), the left arcuate fasciculus and the left middle longitudinal fasciculus (MdLF) were identified in the connectometry analysis of manipulable objects. The arcuate fasciculus was specifically associated with manipulable objects, as it was not identified in the connectometry analysis of places (see Fig. 5). (**B**) There is convergence between the VLAM-identified aIPS and SMG lesion site (see Fig. 3) and the connectometry-identified left arcuate fasciculus (axial slice *Z* = 30). Inferior to that is a lesion site in the posterior middle temporal gyrus that overlaps with the left IFOF, the left ILF, and the left MdLF.

The connectometry analysis of manipulable objects identified a pathway connecting the left ventral OTC to VLAM-identified lesion sites via the left inferior fronto-occipital fasciculus, the left inferior longitudinal fasciculus, the left arcuate fasciculus and the left middle longitudinal fasciculus (Fig. 4A; Table 2). There was convergence between the VLAM-identified left SMG and aIPS (Fig. 4B, red) and the connectometry-identified left arcuate fasciculus (Fig. 4B, yellow; axial slice *Z* = 30). Inferior to that site is a second area in which the VLAM-identified lesion site in the posterior middle temporal gyrus overlaps with the connectometry-identified inferior fronto-occipital fasciculus (Fig. 4B, green), inferior longitudinal fasciculus (Fig. 4B, blue) and middle longitudinal fasciculus (Fig. 4B, purple). See Supplementary Fig. 6 for confirmation that these effects are not related to variability in lesion aetiology.

**Table 2:** White matter fibre pathways identified in the connectometry analysis of manipulable objects and places

Analysis	Tract name	Number of fibres in tract	Composition of tracts identified in the connectometry analysis
Manipulable objects	Inferior fronto-occipital fasciculus	11 551	64%
Manipulable objects	Inferior longitudinal fasciculus	3176	18%
Manipulable objects	Arcuate fasciculus	1537	8%
Manipulable objects	Middle longitudinal fasciculus	1402	8%
Places	Inferior fronto-occipital fasciculus	3114	74%
Places	Middle longitudinal fasciculus	453	11%
Places	Extreme capsule	368	9%
Places	Inferior longitudinal fasciculus	259	6%

DSI Studio was used to determine the percentage of white matter fibres identified that were in each tract. White matter tracts with less than 5% of fibres identified are not reported.

**Figure 5:**
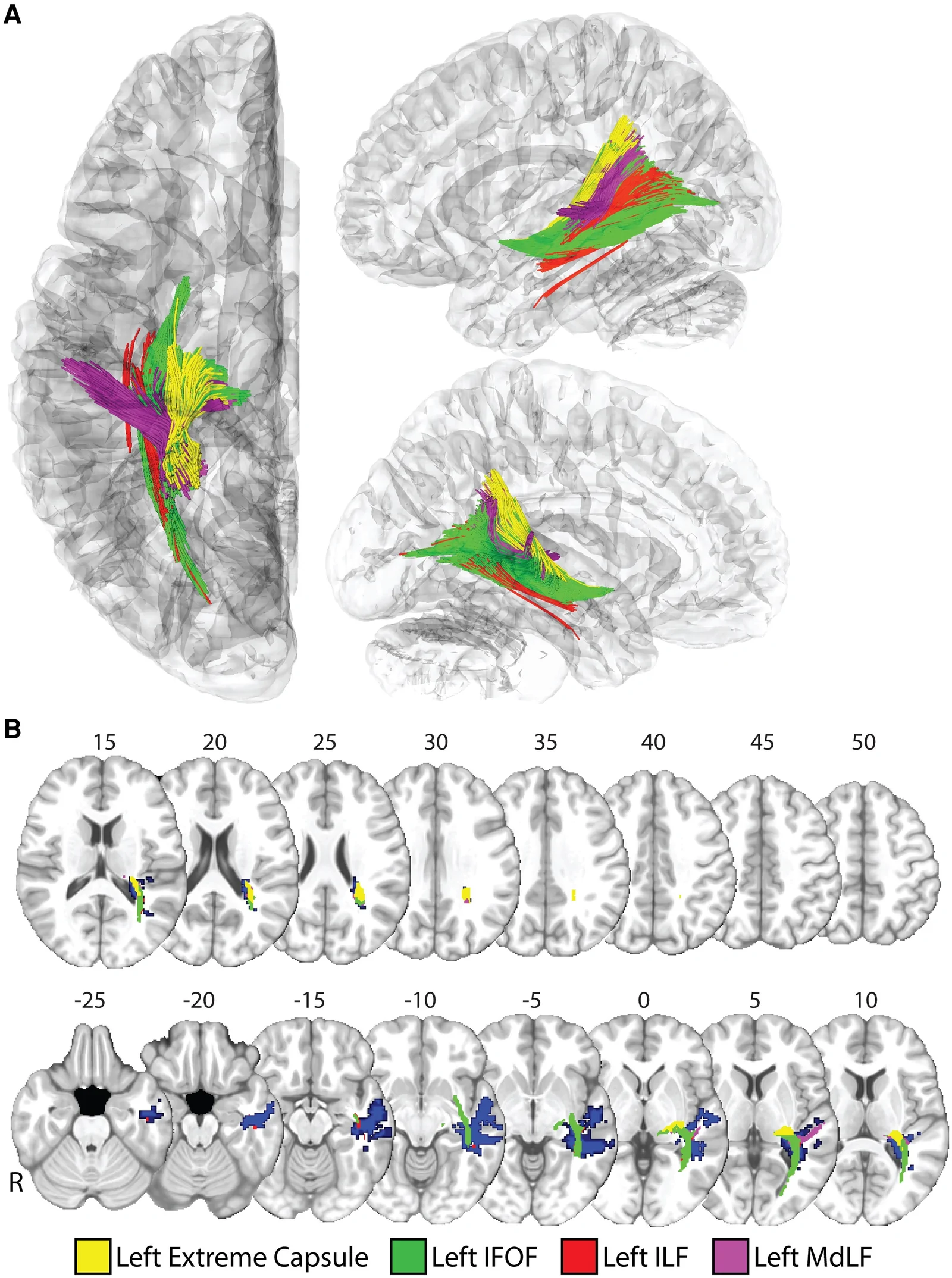
**Connectome-based lesion-activity mapping of places in the left ventral OTC.** This analysis was performed in 75 participants. The resulting fibres represent a single data point in the analysis. (**A**) The connectometry analysis of places identified the left extreme capsule, the left inferior fronto-occipital fasciculus (IFOF), the left inferior longitudinal fasciculus (ILF) and the left middle longitudinal fasciculus (MdLF). (**B**) These white matter tracts overlap with the lesion sites that exhibited a differentially stronger VLAM effect for place stimuli compared to manipulable objects (blue; see Fig. 3).

The connectometry analysis of places identified the left inferior fronto-occipital fasciculus, the left middle longitudinal fasciculus, the left extreme capsule and the left inferior longitudinal fasciculus (Fig. 5A; Table 2). As with manipulable objects, there was convergence between the VLAM-identified lesion sites for places and the connectometry-identified white matter tracts in the white matter medial to the inferior parietal lobule (Fig. 5B, axial slices *Z* = 15–30) and in the left superior, middle, inferior temporal gyri and the white matter lateral to the left hippocampus (Fig. 5B, axial slices *Z* = −25 to 10).

In summary, the inferior fronto-occipital fasciculus, inferior longitudinal fasciculus and the middle longitudinal fasciculus were identified in the connectometry analyses for both manipulable objects and places (see Figs 4 and 5). By contrast, the descending portion of the left arcuate fasciculus was related to reductions in fMRI responses to manipulable objects, and not to places, in left ventral OTC.

## Discussion

We found that lesions to the left SMG and aIPS, and the white matter undercutting the LIPL (Fig. 2), were associated with reduced BOLD responses for visual processing of manipulable objects in left ventral OTC. We also found that the descending portion of the arcuate fasciculus is a key white matter pathway mediating the distal effect of parietal lesions on left ventral OTC responses. We used BOLD responses to place stimuli in left ventral OTC as a within-participant baseline to avoid confounds caused by between-participant differences. For instance, differences in the speed with which tumours grow could modulate the degree to which they induce functional reorganization in the brain—such factors are, while critically important to understand, unable to explain the specificity, for manipulable objects, of the effect we have reported. Collectively, our results demonstrate that lesions to parietal action representations cause reductions to BOLD responses distal to the site of injury in the left ventral OTC and which are specific to processing of manipulable objects. This general phenomenon was first described in the language system by Price and colleagues (2001), who referred to it as dynamic diaschisis^86^ (see also Carrera and Tononi^87^ and Campos *et al*.^88^).

### Voxel-based lesion-activity mapping results

The VLAM analysis of manipulable objects in left ventral OTC also identified the posterior middle temporal gyrus, which has been argued to support conceptual processing of manipulable objects.^32,40,89^ These findings support the inference that the LIPL, the left posterior middle temporal gyrus and the collateral sulcus form a network, among other regions, that supports functional object use. In line with that view, reduced structural integrity of the left arcuate fasciculus, a tract mediating structural connectivity between the LIPL and left posterior middle temporal gyrus,^54^ was associated with reduced manipulable object preferences in left ventral OTC.

Interactions between the dorsal and ventral streams, and more broadly between parietal and OTC regions, are reciprocal.^14^ Additional research is needed to test the conditions under which object processing in the ventral stream modulates dorsal stream processing of the action target. We did not have ventral OTC lesion coverage within our sample, and thus were unable to test how ventral OTC lesions affect parietal representations of manipulable objects (see Supplementary Fig. 7). However, other findings suggest there are substantial inputs to inferior parietal regions from OTC object representations (for review of fMRI and neuropsychological findings, see Mahon and Almeida^14^). The dorsal stream has access to proprietary visual representations of the visual form^90-93^ and location of an object with respect to the body.^94^ Those dorsal visual object representations define volumetric and spatial properties of grasp targets, but ultimately, functional object grasping requires inputs from the ventral stream about the conceptual and perceptual attributes of the object.^13^ One of the simplest observations to support that inference is compelling: the classic ventral-stream-lesioned patient DF, who grasps objects in a volumetrically and biomechanically appropriate manner despite a dense visual form agnosia. Carey and colleagues (1996) found that, although DF’s intact dorsal stream is sufficient to support volumetric grasping, she does not display end-state comfort. DF’s grasp planning could not anticipate action-relevant properties that depend on object identity, such as how the object could be used for functional manipulation.^95^ Such observations indicate that an intact ventral stream is required for functional object grasping and use (for discussion, see Goodale *et al*.^8,11^ and Milner and Goodale^96^).

A key issue motivated by our finding is to better understand how the dependence of ventral OTC responses on left inferior parietal processing relates to clinical and experimental measures of object-directed action. For instance, parallel investigations of individuals with left fronto-parietal stroke lesions could test whether recovery from upper limb apraxia is linked to establishment of functional connectivity between the right parietal lobule and left ventral OTC.^97,98^ This may involve reorganization of object-directed action planning and processing across the hemispheres.

Another important direction is to investigate object-directed action in relation to fibre tract integrity of the left arcuate fasciculus, as well as the IPS-FG fibre tract, a white matter pathway coursing vertically, posterior to the arcuate fasciculus and anterior to the vertical occipital fasciculus, and which connects the posterior IPS to the fusiform gyrus.^99-102^ Future studies leveraging causal evidence from white matter dissection mapping will be crucial to advance understanding of the structural basis of functional diaschisis between parietal and OTC regions.

As a first step, we carried out preliminary analyses relating the strength of manipulable object preferences in the left ventral OTC to participants’ ability to gesture object use. Object use gesturing data were available in 23 participants with lesions. We found that participants who made spatial errors during object use gesturing exhibited reduced BOLD responses for manipulable objects in the left ventral OTC, compared to participants who do not make errors (*z* = −2.36, *P* < 0.05; Supplementary Fig. 8). We emphasize that these results must be taken as preliminary, as the reduced sample size prevents robust conclusions. Nonetheless, it is interesting that spatial errors—which include incorrect configurations of the fingers, hand and arm—are associated with reduced BOLD responses in left ventral OTC during visual processing of manipulable objects.

### Connectometry results

The connectometry analysis of both manipulable objects and places identified the left inferior fronto-occipital fasciculus, the left inferior longitudinal fasciculus and the left middle longitudinal fasciculus. Whereas structural integrity of the left inferior fronto-occipital fasciculus has been found to predict tool use accuracy in patients with lesions,^59^ the inferior and middle longitudinal fasciculi have not been implicated in prior studies of object-directed action. One possibility is that these pathways integrate processing across the hierarchy of visual regions to support both feedforward and feedback processing, irrespective of the stimulus content. By contrast, the integrity of the descending portion of the left arcuate fasciculus was related to fMRI responses to manipulable objects, but not places, in left ventral OTC. Human white matter dissection studies have shown that a portion of the posterior descending segment of the left arcuate fasciculus terminates in the posterior middle temporal gyrus^103^ (Fig. 4, yellow). Collectively, our findings support the inference that BOLD responses in left ventral OTC are modulated both by processes that result from a bottom-up analysis of the visual input within the ventral stream and by inputs from parietal action areas, mediated in part by the descending segment of the left arcuate fasciculus.

## Conclusion

Action-relevant computations supporting functional object grasping and manipulation provide inputs that drive analysis of material properties of objects in left ventral OTC. Action plans include predictions about how an action will look and feel when produced. As the action is planned and starts to unfold, real-time analysis of surface texture and material composition in left ventral OTC can update the current and future state of the hand and arm.^14,15,22^ Evidence from direct electrical stimulation studies during awake brain mapping demonstrates that stimulation of the left SMG and aIPS can cause disruptions in hand shaping for object manipulation^104,105^ (see also Binkofski *et al*.^106^). Lesion-symptom mapping studies also indicate that lesions to the left SMG and left posterior middle temporal gyrus are associated with impaired object use gesturing.^33,107-112^

Ultimately, actions towards objects are based on an understanding of what will be done with the object to satisfy behavioural goals. Dorsal stream visuomotor processing occurs in parallel to processing within the ventral stream^10,113-115^ and may serve to bias processing within the ventral stream to extract visual form, surface texture and materials properties to facilitate functional grasping and object manipulation in the service of behavioural goals. Our proposal is that an initial low-resolution representation of a visual stimulus as an action target (via the dorsal stream) triggers finer-grained analyses integrating surface texture and material properties (OTC), with visual shape (lateral occipital-temporal cortex) and with conceptual representations (posterior middle temporal gyrus) in support of the action plan. Integration of object material properties into the action plan is a key computational goal that could be achieved by the parietal-to-OTC connectivity we have described here.

## Supplementary Material

fcag293_Supplementary_Data

## Data Availability

The whole-brain maps of contrast-weighted *t*-values, lesions in MNI space, ROIs localized and analysis code are available via GitHub (https://github.com/frankgarcea/GarceaColleagues_BrainComms.git). Individuals interested in raw MRI data should contact the corresponding author to complete a data use agreement, as per the IRB office at the University of Rochester.

## References

[1] Garcea FE, Mahon BZ. Parcellation of left parietal tool representations by functional connectivity. Neuropsychologia. 2014;60:131–143. 24892224 10.1016/j.neuropsychologia.2014.05.018 PMC4116796

[2] Gallivan JP, McLean DA, Valyear KF, Culham JC. Decoding the neural mechanisms of human tool use. Elife. 2013;2:e00425. 23741616 10.7554/eLife.00425 PMC3667577

[3] Reynaud E, Lesourd M, Navarro J, Osiurak F. On the neurocognitive origins of human tool use: A critical review of neuroimaging data. Neurosci Biobehav Rev. 2016;64:421–437. 26976352 10.1016/j.neubiorev.2016.03.009

[4] Buxbaum LJ . Learning, remembering, and predicting how to use tools: Distributed neurocognitive mechanisms: Comment on Osiurak and Badets (2016). Psychol Rev. 2017;124(3):346–360. 28358565 10.1037/rev0000051 PMC5375056

[5] Osiurak F, Federico G, Fournel A, et al Shaping the physical world to our ends through the left PF technical-cognition area. Elife. 2025;13:RP94578. 40243287 10.7554/eLife.94578 PMC12005713

[6] Orban GA, Caruana F. The neural basis of human tool use. Front Psychol. 2014;5:310. 24782809 10.3389/fpsyg.2014.00310 PMC3988392

[7] Almeida J, Mahon BZ, Nakayama K, Caramazza A. Unconscious processing dissociates along categorical lines. Proc Natl Acad Sci U S A. 2008;105(39):15214–15218. 18809923 10.1073/pnas.0805867105 PMC2567517

[8] Goodale MA, Milner AD, Jakobson LS, Carey DP. A neurological dissociation between perceiving objects and grasping them. Nature. 1991;349(6305):154–156. 1986306 10.1038/349154a0

[9] Binkofski F, Dohle C, Posse S, et al Human anterior intraparietal area subserves prehension: A combined lesion and functional MRI activation study. Neurology. 1998;50(5):1253–1259. 9595971 10.1212/wnl.50.5.1253

[10] Rizzolatti G, Matelli M. Two different streams form the dorsal visual system: Anatomy and functions. Exp Brain Res. 2003;153(2):146–157. 14610633 10.1007/s00221-003-1588-0

[11] Goodale MA, Jakobson LS, Keillor JM. Differences in the visual control of pantomimed and natural grasping movements. Neuropsychologia. 1994;32(10):1159–1178. 7845558 10.1016/0028-3932(94)90100-7

[12] Creem SH, Proffitt DR. Grasping objects by their handles: A necessary interaction between cognition and action. J Exp Psychol Hum Percept Perform. 2001;27(1):218–228. 11248935 10.1037//0096-1523.27.1.218

[13] Rosenbaum DA, Marchak F, Barnes HJ, Vaughan J, Slotta JD, Jorgensen MJ. Constraints for action selection: Overhand versus underhand grips. In: Jeannerod M, ed. Attention and performance XIII: Motor representation and control. Erlbaum; 1990:321–342.

[14] Mahon BZ, Almeida J. Reciprocal interactions among parietal and occipito-temporal representations support everyday object-directed actions. Neuropsychologia. 2024;198:108841. 38430962 10.1016/j.neuropsychologia.2024.108841 PMC11498102

[15] Garcea FE, Almeida J, Sims MH, et al Domain-specific diaschisis: Lesions to parietal action areas modulate neural responses to tools in the ventral stream. Cereb Cortex. 2019;29(7):3168–3181. 30169596 10.1093/cercor/bhy183 PMC6933536

[16] Gallivan JP, Chapman CS, McLean DA, Flanagan JR, Culham JC. Activity patterns in the category-selective occipitotemporal cortex predict upcoming motor actions. Eur J Neurosci. 2013;38(3):2408–2424. 23581683 10.1111/ejn.12215

[17] Almeida J, Fintzi AR, Mahon BZ. Tool manipulation knowledge is retrieved by way of the ventral visual object processing pathway. Cortex. 2013;49(9):2334–2344. 23810714 10.1016/j.cortex.2013.05.004 PMC3795789

[18] Buxbaum LJ, Randerath J. Limb apraxia and the left parietal lobe. Handb Clin Neurol. 2018;151:349–363. 29519468 10.1016/B978-0-444-63622-5.00017-6 PMC8139361

[19] Hutchison RM, Culham JC, Everling S, Flanagan JR, Gallivan JP. Distinct and distributed functional connectivity patterns across cortex reflect the domain-specific constraints of object, face, scene, body, and tool category-selective modules in the ventral visual pathway. Neuroimage. 2014;96:216–236. 24699018 10.1016/j.neuroimage.2014.03.068

[20] Rounis E, Binkofski F. Limb apraxias: The influence of higher order perceptual and semantic deficits in motor recovery after stroke. Stroke. 2023;54(1):30–43. 36542070 10.1161/STROKEAHA.122.037948

[21] Yang H, He C, Han Z, Bi Y. Domain-specific functional coupling between dorsal and ventral systems during action perception. Sci Rep. 2020;10(1):21200. 33273681 10.1038/s41598-020-78276-4 PMC7713359

[22] Gallivan JP, Culham JC. Neural coding within human brain areas involved in actions. Curr Opin Neurobiol. 2015;33:141–149. 25876179 10.1016/j.conb.2015.03.012

[23] Catani M, Jones DK, Donato R, Ffytche DH. Occipito-temporal connections in the human brain. Brain. 2003;126(Pt 9):2093–2107. 12821517 10.1093/brain/awg203

[24] Forkel SJ, Thiebaut de Schotten M, Kawadler JM, Dell'Acqua F, Danek A, Catani M. The anatomy of fronto-occipital connections from early blunt dissections to contemporary tractography. Cortex. 2014;56:73–84. 23137651 10.1016/j.cortex.2012.09.005

[25] Giampiccolo D, Herbet G, Duffau H. The inferior fronto-occipital fasciculus: Bridging phylogeny, ontogeny and functional anatomy. Brain. 2025;148:1507–1525. 39932875 10.1093/brain/awaf055 PMC12074009

[26] DiCarlo JJ, Zoccolan D, Rust NC. How does the brain solve visual object recognition? Neuron. 2012;73(3):415–434. 22325196 10.1016/j.neuron.2012.01.010 PMC3306444

[27] Riesenhuber M, Poggio T. Models of object recognition. Nat Neurosci. 2000;3(11):1199–1204. 11127838 10.1038/81479

[28] Op de Beeck HP, Haushofer J, Kanwisher NG. Interpreting fMRI data: Maps, modules and dimensions. Nat Rev Neurosci. 2008;9(2):123–135. 18200027 10.1038/nrn2314 PMC2731480

[29] Ungerleider LG, Mishkin M. Two cortical visual systems. In: Ingle DA, Goodale MA, Mansfield RJW, eds. Analysis of visual behavior. MIT Press; 1982:chap 549–586.

[30] Brandi ML, Wohlschlager A, Sorg C, Hermsdorfer J. The neural correlates of planning and executing actual tool use. J Neurosci. 2014;34(39):13183–13194. 25253863 10.1523/JNEUROSCI.0597-14.2014 PMC6608341

[31] Frey SH . What puts the how in where? Tool use and the divided visual streams hypothesis. Cortex. 2007;43(3):368–375. 17533760 10.1016/s0010-9452(08)70462-3

[32] Chao LL, Haxby JV, Martin A. Attribute-based neural substrates in temporal cortex for perceiving and knowing about objects. Nat Neurosci. 1999;2(10):913–919. 10491613 10.1038/13217

[33] Mahon BZ, Milleville SC, Negri GA, Rumiati RI, Caramazza A, Martin A. Action-related properties shape object representations in the ventral stream. Neuron. 2007;55(3):507–520. 17678861 10.1016/j.neuron.2007.07.011 PMC2000824

[34] Allison T, McCarthy G, Nobre A, Puce A, Belger A. Human extrastriate visual cortex and the perception of faces, words, numbers, and colors. Cereb Cortex. 1994;4(5):544–554. 7833655 10.1093/cercor/4.5.544

[35] Martin A, Wiggs CL, Ungerleider LG, Haxby JV. Neural correlates of category-specific knowledge. Nature. 1996;379(6566):649–652. 8628399 10.1038/379649a0

[36] Kanwisher N, McDermott J, Chun MM. The fusiform face area: A module in human extrastriate cortex specialized for face perception. J Neurosci. 1997;17(11):4302–4311. 9151747 10.1523/JNEUROSCI.17-11-04302.1997 PMC6573547

[37] Downing PE, Jiang Y, Shuman M, Kanwisher N. A cortical area selective for visual processing of the human body. Science. 2001;293(5539):2470–2473. 11577239 10.1126/science.1063414

[38] Cohen L, Lehericy S, Chochon F, Lemer C, Rivaud S, Dehaene S. Language-specific tuning of visual cortex? Functional properties of the visual word form area. Brain. 2002;125(Pt 5):1054–1069. 11960895 10.1093/brain/awf094

[39] Grill-Spector K, Henson R, Martin A. Repetition and the brain: Neural models of stimulus-specific effects. Trends Cogn Sci. 2006;10(1):14–23. 16321563 10.1016/j.tics.2005.11.006

[40] Martin A . The representation of object concepts in the brain. Annu Rev Psychol. 2007;58:25–45. 16968210 10.1146/annurev.psych.57.102904.190143

[41] Peelen MV, Downing PE. The neural basis of visual body perception. Nat Rev Neurosci. 2007;8(8):636–648. 17643089 10.1038/nrn2195

[42] Epstein R, Kanwisher N. A cortical representation of the local visual environment. Nature. 1998;392(6676):598–601. 9560155 10.1038/33402

[43] Bar M, Aminoff E. Cortical analysis of visual context. Neuron. 2003;38(2):347–358. 12718867 10.1016/s0896-6273(03)00167-3

[44] Chen Q, Garcea FE, Almeida J, Mahon BZ. Connectivity-based constraints on category-specificity in the ventral object processing pathway. Neuropsychologia. 2017;105:184–196. 27876509 10.1016/j.neuropsychologia.2016.11.014 PMC5438294

[45] Downing PE, Chan AW, Peelen MV, Dodds CM, Kanwisher N. Domain specificity in visual cortex. Cereb Cortex. 2006;16(10):1453–1461. 16339084 10.1093/cercor/bhj086

[46] Stevens WD, Tessler MH, Peng CS, Martin A. Functional connectivity constrains the category-related organization of human ventral occipitotemporal cortex. Hum Brain Mapp. 2015;36(6):2187–2206. 25704493 10.1002/hbm.22764 PMC4414790

[47] Hutchison RM, Gallivan JP. Functional coupling between frontoparietal and occipitotemporal pathways during action and perception. Cortex. 2018;98:8–27. 27890325 10.1016/j.cortex.2016.10.020

[48] Garcea FE, Chen Q, Vargas R, Narayan DA, Mahon BZ. Task- and domain-specific modulation of functional connectivity in the ventral and dorsal object-processing pathways. Brain Struct Funct. 2018;223(6):2589–2607. 29536173 10.1007/s00429-018-1641-1 PMC6252262

[49] Cavina-Pratesi C, Kentridge RW, Heywood CA, Milner AD. Separate processing of texture and form in the ventral stream: Evidence from FMRI and visual agnosia. Cereb Cortex. 2010;20(2):433–446. 19478035 10.1093/cercor/bhp111

[50] Stasenko A, Garcea FE, Dombovy M, Mahon BZ. When concepts lose their color: A case of object-color knowledge impairment. Cortex. 2014;58:217–238. 25058612 10.1016/j.cortex.2014.05.013 PMC4135534

[51] Cant JS, Goodale MA. Attention to form or surface properties modulates different regions of human occipitotemporal cortex. Cereb Cortex. 2007;17(3):713–731. 16648452 10.1093/cercor/bhk022

[52] Cant JS, Arnott SR, Goodale MA. fMR-adaptation reveals separate processing regions for the perception of form and texture in the human ventral stream. Exp Brain Res. 2009;192(3):391–405. 18815774 10.1007/s00221-008-1573-8

[53] Wolk DA, Coslett HB, Glosser G. The role of sensory-motor information in object recognition: Evidence from category-specific visual agnosia. Brain Lang. 2005;94(2):131–146. 15896389 10.1016/j.bandl.2004.10.015

[54] Ramayya AG, Glasser MF, Rilling JK. A DTI investigation of neural substrates supporting tool use. Cereb Cortex. 2010;20(3):507–516. 19608779 10.1093/cercor/bhp141

[55] Panesar SS, Belo JTA, Yeh FC, Fernandez-Miranda JC. Structure, asymmetry, and connectivity of the human temporo-parietal aslant and vertical occipital fasciculi. Brain Struct Funct. 2019;224(2):907–923. 30542766 10.1007/s00429-018-1812-0 PMC7026858

[56] Budisavljevic S, Dell'Acqua F, Castiello U. Cross-talk connections underlying dorsal and ventral stream integration during hand actions. Cortex. 2018;103:224–239. 29660652 10.1016/j.cortex.2018.02.016

[57] Yeatman JD, Weiner KS, Pestilli F, Rokem A, Mezer A, Wandell BA. The vertical occipital fasciculus: A century of controversy resolved by in vivo measurements. Proc Natl Acad Sci U S A. 2014;111(48):E5214–E5223. 25404310 10.1073/pnas.1418503111 PMC4260539

[58] Makris N, Preti MG, Asami T, et al Human middle longitudinal fascicle: Variations in patterns of anatomical connections. Brain Struct Funct. 2013;218(4):951–968. 22782432 10.1007/s00429-012-0441-2 PMC3500586

[59] Bi Y, Han Z, Zhong S, et al The white matter structural network underlying human tool use and tool understanding. J Neurosci. 2015;35(17):6822–6835. 25926458 10.1523/JNEUROSCI.3709-14.2015 PMC6605181

[60] Chen Q, Garcea FE, Jacobs RA, Mahon BZ. Abstract representations of object-directed action in the left inferior parietal lobule. Cereb Cortex. 2018;28(6):2162–2174. 28605410 10.1093/cercor/bhx120 PMC6019004

[61] Garcea FE, Kristensen S, Almeida J, Mahon BZ. Resilience to the contralateral visual field bias as a window into object representations. Cortex. 2016;81:14–23. 27160998 10.1016/j.cortex.2016.04.006 PMC4958537

[62] Chen Q, Garcea FE, Mahon BZ. The representation of object-directed action and function knowledge in the human brain. Cereb Cortex. 2016;26(4):1609–1618. 25595179 10.1093/cercor/bhu328 PMC4785951

[63] Erdogan G, Chen Q, Garcea FE, Mahon BZ, Jacobs RA. Multisensory part-based representations of objects in human lateral occipital Cortex. J Cogn Neurosci. 2016;28(6):869–881. 26918587 10.1162/jocn_a_00937 PMC5203770

[64] Kristensen S, Garcea FE, Mahon BZ, Almeida J. Temporal frequency tuning reveals interactions between the dorsal and ventral visual streams. J Cogn Neurosci. 2016;28(9):1295–1302. 27082048 10.1162/jocn_a_00969 PMC5193157

[65] Oldfield RC . The assessment and analysis of handedness: The Edinburgh inventory. Neuropsychologia. 1971;9(1):97–113. 5146491 10.1016/0028-3932(71)90067-4

[66] Schwarzbach J . A simple framework (ASF) for behavioral and neuroimaging experiments based on the psychophysics toolbox for MATLAB. Behav Res Methods. 2011;43(4):1194–1201. 21614662 10.3758/s13428-011-0106-8

[67] Pelli DG . The VideoToolbox software for visual psychophysics: Transforming numbers into movies. Spat Vis. 1997;10(4):437–442. 9176953

[68] Peirce JW . Psychopy–psychophysics software in python. J Neurosci Methods. 2007;162(1–2):8–13. 17254636 10.1016/j.jneumeth.2006.11.017 PMC2018741

[69] Brett M, Leff AP, Rorden C, Ashburner J. Spatial normalization of brain images with focal lesions using cost function masking. Neuroimage. 2001;14(2):486–500. 11467921 10.1006/nimg.2001.0845

[70] Metzgar R, Stoll H, Grafton ST, Buxbaum LJ, Garcea FE. Single-case disconnectome lesion-symptom mapping: Identifying two subtypes of limb apraxia. Neuropsychologia. 2022;170:108210. 35283160 10.1016/j.neuropsychologia.2022.108210 PMC9189785

[71] Esteban O, Markiewicz CJ, Blair RW, et al fMRIPrep: A robust preprocessing pipeline for functional MRI. Nat Methods. 2019;16(1):111–116. 30532080 10.1038/s41592-018-0235-4 PMC6319393

[72] Kriegeskorte N, Simmons WK, Bellgowan PS, Baker CI. Circular analysis in systems neuroscience: The dangers of double dipping. Nat Neurosci. 2009;12(5):535–540. 19396166 10.1038/nn.2303 PMC2841687

[73] DeMarco AT, Turkeltaub PE. A multivariate lesion symptom mapping toolbox and examination of lesion-volume biases and correction methods in lesion-symptom mapping. Hum Brain Mapp. 2018;39(11):4169–4182. 29972618 10.1002/hbm.24289 PMC6647024

[74] Garcea FE, Buxbaum LJ. Mechanisms and neuroanatomy of response selection in tool and non-tool action tasks: Evidence from left-hemisphere stroke. Cortex. 2023;167:335–350. 37598647 10.1016/j.cortex.2023.06.012 PMC10543550

[75] Garcea FE, Greene C, Grafton ST, Buxbaum LJ. Structural disconnection of the tool use network after left hemisphere stroke predicts limb apraxia severity. Cereb Cortex Commun. 2020;1(1):tgaa035. 33134927 10.1093/texcom/tgaa035 PMC7573742

[76] Garcea FE, Stoll H, Buxbaum LJ. Reduced competition between tool action neighbors in left hemisphere stroke. Cortex. 2019;120:269–283. 31352237 10.1016/j.cortex.2019.05.021 PMC6951425

[77] Lacey EH, Skipper-Kallal LM, Xing S, Fama ME, Turkeltaub PE. Mapping common aphasia assessments to underlying cognitive processes and their neural substrates. Neurorehabil Neural Repair. 2017;31(5):442–450. 28135902 10.1177/1545968316688797 PMC5393922

[78] Skipper-Kallal LM, Lacey EH, Xing S, Turkeltaub PE. Functional activation independently contributes to naming ability and relates to lesion site in post-stroke aphasia. Hum Brain Mapp. 2017;38(4):2051–2066. 28083891 10.1002/hbm.23504 PMC6867020

[79] Binder JR, Desai RH. The neurobiology of semantic memory. Trends Cogn Sci. 2011;15(11):527–536. 22001867 10.1016/j.tics.2011.10.001 PMC3350748

[80] Binder JR, Desai RH, Graves WW, Conant LL. Where is the semantic system? A critical review and meta-analysis of 120 functional neuroimaging studies. Cereb Cortex. 2009;19(12):2767–2796. 19329570 10.1093/cercor/bhp055 PMC2774390

[81] Kuhnke P, Chapman CA, Cheung VKM, et al The role of the angular gyrus in semantic cognition: A synthesis of five functional neuroimaging studies. Brain Struct Funct. 2023;228(1):273–291. 35476027 10.1007/s00429-022-02493-y PMC9813249

[82] Kravitz DJ, Saleem KS, Baker CI, Mishkin M. A new neural framework for visuospatial processing. Nat Rev Neurosci. 2011;12(4):217–230. 21415848 10.1038/nrn3008 PMC3388718

[83] Yeh FC, Badre D, Verstynen T. Connectometry: A statistical approach harnessing the analytical potential of the local connectome. Neuroimage. 2016;125:162–171. 26499808 10.1016/j.neuroimage.2015.10.053

[84] Yourganov G, Fridriksson J, Rorden C, Gleichgerrcht E, Bonilha L. Multivariate connectome-based symptom mapping in post-stroke patients: Networks supporting language and speech. J Neurosci. 2016;36(25):6668–6679. 27335399 10.1523/JNEUROSCI.4396-15.2016 PMC4916245

[85] Gleichgerrcht E, Fridriksson J, Rorden C, Bonilha L. Connectome-based lesion-symptom mapping (CLSM): A novel approach to map neurological function. Neuroimage Clin. 2017;16:461–467. 28884073 10.1016/j.nicl.2017.08.018 PMC5581860

[86] Price CJ, Warburton EA, Moore CJ, Frackowiak RS, Friston KJ. Dynamic diaschisis: Anatomically remote and context-sensitive human brain lesions. J Cogn Neurosci. 2001;13(4):419–429. 11388916 10.1162/08989290152001853

[87] Carrera E, Tononi G. Diaschisis: Past, present, future. Brain. 2014;137(Pt 9):2408–2422. 24871646 10.1093/brain/awu101

[88] Campos B, Choi H, DeMarco AT, et al Rethinking remapping: Circuit mechanisms of recovery after stroke. J Neurosci. 2023;43(45):7489–7500. 37940595 10.1523/JNEUROSCI.1425-23.2023 PMC10634578

[89] Garcea FE, Buxbaum LJ. Gesturing tool use and tool transport actions modulates inferior parietal functional connectivity with the dorsal and ventral object processing pathways. Hum Brain Mapp. 2019;40(10):2867–2883. 30900321 10.1002/hbm.24565 PMC6548598

[90] Konen CS, Kastner S. Two hierarchically organized neural systems for object information in human visual cortex. Nat Neurosci. 2008;11(2):224–231. 18193041 10.1038/nn2036

[91] Freud E, Ganel T, Shelef I, Hammer MD, Avidan G, Behrmann M. Three-dimensional representations of objects in dorsal Cortex are dissociable from those in ventral Cortex. Cereb Cortex. 2017;27(1):422–434. 26483400 10.1093/cercor/bhv229 PMC13100970

[92] Almeida J, Mahon BZ, Zapater-Raberov V, et al Grasping with the eyes: The role of elongation in visual recognition of manipulable objects. Cogn Affect Behav Neurosci. 2014;14(1):319–335. 23996788 10.3758/s13415-013-0208-0

[93] Chen J, Snow JC, Culham JC, Goodale MA. What role does “elongation” play in “tool-specific” activation and connectivity in the dorsal and ventral visual streams? Cereb Cortex. 2018;28(4):1117–1131. 28334063 10.1093/cercor/bhx017 PMC6454576

[94] Freud E, Robinson AK, Behrmann M. More than action: The dorsal pathway contributes to the perception of 3-D structure. J Cogn Neurosci. 2018;30(7):1047–1058. 29561234 10.1162/jocn_a_01262

[95] Carey DP, Harvey M, Milner AD. Visuomotor sensitivity for shape and orientation in a patient with visual form agnosia. Neuropsychologia. 1996;34(5):329–337. 9148189 10.1016/0028-3932(95)00169-7

[96] Milner AD, Goodale MA. Two visual systems re-viewed. Neuropsychologia. 2008;46(3):774–785. 18037456 10.1016/j.neuropsychologia.2007.10.005

[97] Watson CE, Gotts SJ, Martin A, Buxbaum LJ. Bilateral functional connectivity at rest predicts apraxia after left hemisphere stroke. Neuroimage Clin. 2019;21:101526. 30612063 10.1016/j.nicl.2018.08.033 PMC6319198

[98] Dressing A, Kaller CP, Martin M, et al Anatomical correlates of recovery in apraxia: A longitudinal lesion-mapping study in stroke patients. Cortex. 2021;142:104–121. 34265734 10.1016/j.cortex.2021.06.001

[99] Bullock D, Takemura H, Caiafa CF, et al Associative white matter connecting the dorsal and ventral posterior human cortex. Brain Struct Funct. 2019;224(8):2631–2660. 31342157 10.1007/s00429-019-01907-8

[100] Jitsuishi T, Hirono S, Yamamoto T, Kitajo K, Iwadate Y, Yamaguchi A. White matter dissection and structural connectivity of the human vertical occipital fasciculus to link vision-associated brain cortex. Sci Rep. 2020;10(1):820. 31965011 10.1038/s41598-020-57837-7 PMC6972933

[101] Borra E, Luppino G. Functional anatomy of the macaque temporo-parieto-frontal connectivity. Cortex. 2016;97:306–326. 28041615 10.1016/j.cortex.2016.12.007

[102] Catani M, Robertsson N, Beyh A, et al Short parietal lobe connections of the human and monkey brain. Cortex. 2017;97:339–357. 29157936 10.1016/j.cortex.2017.10.022

[103] Giampiccolo D, Duffau H. Controversy over the temporal cortical terminations of the left arcuate fasciculus: A reappraisal. Brain. 2022;145(4):1242–1256. 35142842 10.1093/brain/awac057

[104] Fornia L, Leonetti A, Puglisi G, et al The parietal architecture binding cognition to sensorimotor integration: A multimodal causal study. Brain. 2024;147(1):297–310. 37715997 10.1093/brain/awad316 PMC10766244

[105] Fornia L, Rossi M, Rabuffetti M, et al Motor impairment evoked by direct electrical stimulation of human parietal cortex during object manipulation. Neuroimage. 2022;248:118839. 34963652 10.1016/j.neuroimage.2021.118839

[106] Binkofski F, Buccino G, Stephan KM, Rizzolatti G, Seitz RJ, Freund HJ. A parieto-premotor network for object manipulation: Evidence from neuroimaging. Exp Brain Res. 1999;128(1–2):210–213. 10473761 10.1007/s002210050838

[107] Manuel AL, Radman N, Mesot D, et al Inter- and intrahemispheric dissociations in ideomotor apraxia: A large-scale lesion-symptom mapping study in subacute brain-damaged patients. Cereb Cortex. 2013;23(12):2781–2789. 22989580 10.1093/cercor/bhs280

[108] Buxbaum LJ, Shapiro AD, Coslett HB. Critical brain regions for tool-related and imitative actions: A componential analysis. Brain. 2014;137(Pt 7):1971–1985. 24776969 10.1093/brain/awu111 PMC4065019

[109] Weiss PH, Ubben SD, Kaesberg S, et al Where language meets meaningful action: A combined behavior and lesion analysis of aphasia and apraxia. Brain Struct Funct. 2016;221(1):563–576. 25352157 10.1007/s00429-014-0925-3

[110] Hoeren M, Kummerer D, Bormann T, et al Neural bases of imitation and pantomime in acute stroke patients: Distinct streams for praxis. Brain. 2014;137(Pt 10):2796–2810. 25062694 10.1093/brain/awu203

[111] Dressing A, Kaller CP, Nitschke K, et al Neural correlates of acute apraxia: Evidence from lesion data and functional MRI in stroke patients. Cortex. 2019;120:1–21. 31220613 10.1016/j.cortex.2019.05.005

[112] Martin M, Beume L, Kummerer D, et al Differential roles of ventral and dorsal streams for conceptual and production-related components of tool use in acute stroke patients. Cereb Cortex. 2016;26(9):3754–3771. 26271112 10.1093/cercor/bhv179

[113] Binkofski F, Buxbaum LJ. Two action systems in the human brain. Brain Lang. 2013;127(2):222–229. 22889467 10.1016/j.bandl.2012.07.007 PMC4311762

[114] Konen CS, Mruczek RE, Montoya JL, Kastner S. Functional organization of human posterior parietal cortex: Grasping- and reaching-related activations relative to topographically organized cortex. J Neurophysiol. 2013;109(12):2897–2908. 23515795 10.1152/jn.00657.2012 PMC3680816

[115] Culham JC, Danckert SL, DeSouza JF, Gati JS, Menon RS, Goodale MA. Visually guided grasping produces fMRI activation in dorsal but not ventral stream brain areas. Exp Brain Res. 2003;153(2):180–189. 12961051 10.1007/s00221-003-1591-5

