# Supplementary material for: Visual processing of manipulable objects in the ventral stream is modulated by parietal action systems
Source: Brain Commun. 2026 Jul 27;8(4):fcag293. doi: 10.1093/braincomms/fcag293 (PMC13490872; doi:10.1093/braincomms/fcag293)
Supplement: Supplementary file 1 [file fcag293_supplementary_data.docx]

**Supplemental Online Materials**

**Visual processing of manipulable objects in the ventral stream is modulated by parietal action systems**

Frank E. Garcea^1-3*^, Emma L. Strawderman ^1,2^, William Burns^2^,

Matthew Cotroneo^2^, Steven P. Meyers^2-4^, Tyler Schmidt^2^,

Kevin A. Walter^2^, Webster Pilcher^2^, Bradford Z. Mahon^2,5,6^

1. Department of Neuroscience, University of Rochester Medical Center, USA
2. Department of Neurosurgery, University of Rochester Medical Center, USA
3. Department of Brain & Cognitive Sciences, University of Rochester, USA
4. Department of Imaging Sciences, University of Rochester Medical Center, USA
5. Department of Psychology, Carnegie Mellon University, USA
6. Neuroscience Institute, Carnegie Mellon University, USA

* Corresponding author

**Corresponding Author**

Frank E. Garcea, PhD

Department of Neurosurgery

Department of Neuroscience

Dept. of Brain & Cognitive Sciences

University of Rochester Medical Center

frank_garcea@urmc.rochester.edu

**Category Localizer fMRI Task Design**

Three versions of the category localizer experiment were administered over the 8-year study period. In version A of the category localizer, participants viewed scrambled and intact images of manipulable objects, animals, faces, and places in a miniblock design. There were 12 grayscale photographs per category and 8 exemplars of each stimulus, resulting in 96 images per category, and 384 total images. Phase-scrambled versions of the stimuli were created to serve as a baseline condition. Within each miniblock, 12 stimuli from the same category were presented for 1000 ms each (0 ms interstimulus interval), and 12-second fixation periods were presented between miniblocks. Within each run, 8 miniblocks of intact images and 4 miniblocks of phase-scrambled stimuli were presented with the constraint that a category of objects did not repeat across two successive miniblocks.

In version B, grayscale photographs of manipulable objects, animals, fearful faces, faces with neutral affect, body parts, famous places, and words were used. There were 64 exemplars of fearful faces, neutral faces, and words; for all other categories there were 8 exemplars of each stimulus, resulting 64 images per category, and 448 total images. Within each miniblock, 8 stimuli from the same category were presented for 1000 ms each (0 ms interstimulus interval), and 8-second fixation periods were presented between miniblocks. Within each run, 12 miniblocks of intact images and 2 miniblocks of phase-scrambled stimuli were presented with the constraint that a category of objects did not repeat across two successive miniblocks.

In version C, grayscale and color photographs of single-handed manipulable objects (e.g., hammer), two-handed manipulable objects (e.g., axe), household objects (e.g., book), animals, fearful faces, faces with neutral affect, famous, body parts, famous places, non-famous places, fruits and vegetables, surface textures (e.g., leather), words, and numbers were used. There were 48 exemplars of fearful faces, neutral faces, non-famous places, words, and numbers; for all other categories, there were 6 items and 8 exemplars, resulting in 48 images per category, and 720 total images. Within each miniblock, 6 stimuli from the same category were presented for 1800 ms each (0 ms interstimulus interval). Fixation periods ranging from 4 to 12 seconds (8 seconds on average) were presented between miniblocks. Within each run, 15 miniblocks of intact images and 1 miniblock of phase-scrambled stimuli were presented with the constraint that a category of objects did not repeat across two successive miniblocks.

**MRI Acquisition Parameters**

Whole brain MRI was conducted on a 3-Tesla Siemens MAGNETOM Trio scanner with a 32- or 64-channel head coil at the University of Rochester. High-resolution structural T1 contrast images were acquired using a magnetization-prepared rapid gradient echo pulse sequence at the start of each participant’s scanning session (TR = 2530, TE = 3.44ms, flip angle = 7°, FOV = 256mm, matrix = 256 × 256, 192 1 x 1 x 1mm sagittal left-to-right slices). An echo-planar imaging pulse sequence was used for T2* contrast. In the first 3 participants with lesions and 55 neurotypical participants, the acquisition parameters were: TR = 2000ms, TE = 30ms, flip angle = 90°, FOV = 256 × 256 mm, matrix = 64 × 64, 30 inferior-to-superior axial slices, voxel size = 4 x 4 x 4 mm. In the next 40 participants with lesions, the acquisition parameters were: TR = 2200ms, TE = 30ms, flip angle = 90°, FOV = 256 × 256 mm, matrix = 64 × 64, 33 inferior-to-superior axial slices, voxel size = 4 x 4 x 4mm. In the remaining 66 participants with lesions, the acquisition parameters were upgraded to a multi-band sequence: TR = 2200ms, TE = 30ms, flip angle = 70°, FOV = 256 × 256 mm, matrix = 128 × 128, 90 inferior-to-superior axial slices, voxel size = 2 x 2 x 2 mm. Across all participants, the first 4 volumes of each run were discarded to allow for signal equilibration (4 volumes dropped prior to image acquisition).

Diffusion-weighted MRI was acquired using a single-shot echo-planar sequence (60 diffusion directions with b = 1000 s/mm^2^, 10 images with b = 0 s/mm^2^, TR = 8900 msec, TE = 86 msec, FOV = 256 x 256mm, matrix = 128 x 128, voxel size = 2 x 2 x 2 mm, 70 inferior-to-superior axial slices; anterior-to-posterior phase encoding). A double-echo gradient echo field map sequence (echo time difference = 2.46 msec, EPI dwell time = 0.75 msec) was acquired with the same resolution as the DTI sequence to correct for distortion caused by B0 inhomogeneity.

**Supplemental Table 1.** Demographic information for each participant. *Abbreviations.* DNET, Dysembryoplastic Neuroepithelial Tumor; GBM, glioblastoma multiforme; AVM, arteriovenous malformation; PLNTY, polymorphous low grade neuroepithelial tumor of the young. Contrast-weighted *t*-values in parentheses were localized in the even/odd split-half analysis in 64 participants.

| **A.** Participants with brain lesions | | | | | | | | | |
| --- | --- | --- | --- | --- | --- | --- | --- | --- | --- |
| Cumulative  Participant ID | Age | Diagnosis | Gender | Lesion Volume | | Hemisphere | T>AFP  *t*-values | | P>AFT  *t*-value |
| 1 | 52 | Adenocarcinoma | Female | 3018 | | Left | 1.59 (2.06) | | -0.85 (-0.46) |
| 2 | 73 | GBM | Male | 11349 | | Left | Could not localize ROIs | | |
| 3 | 58 | Adenocarcinoma | Female | 85097 | | Left | 1.14 (0.51) | | 2.55 (0.66) |
| 4 | 58 | Astrocytoma | Female | 363396 | | Left | 1.04 | | 3.79 |
| 5 | 69 | Oligodendroglioma | Female | 99764 | | Left | 3.00 (4.42) | | 0.45 (0.31) |
| 6 | 47 | DNET | Female | 34086 | | Left | -0.49 (0.31) | | 1.19 (2.29) |
| 7 | 69 | GBM | Male | 31102 | | Left | Could not localize ROIs | | |
| 8 | 26 | Astrocytoma | Female | 39900 | | Right | Could not localize ROIs | | |
| 9 | 47 | Oligodendroglioma | Male | 99956 | | Left | 1.21 | | 3.77 |
| 10 | 30 | Astrocytoma | Male | 57742 | | Right | 0.75 | | 1.78 |
| 11 | 29 | Astrocytoma | Male | 20878 | | Left | Could not localize ROIs | | |
| 12 | 58 | GBM | Female | 188060 | | Left | 1.18 | | -3.03 |
| 13 | 57 | GBM | Male | 145300 | | Left | 1.53 (1.78) | | 4.11 (0.56) |
| 14 | 54 | Intracerebral hemorrhage | Male | 130675 | | Left | 1.97 (4.30) | | 7.65 (4.67) |
| 15 | 46 | Oligodendroglioma | Male | 67034 | | Left | Could not localize ROIs | | |
| 16 | 51 | Astrocytoma | Female | 36524 | | Right | 1.54 (1.85) | | 1.10 (1.60) |
| 17 | 27 | Oligodendroglioma | Male | 21384 | | Right | 2.77 (4.41) | | 1.20 (0.84) |
| 18 | 25 | Cavernous malformation | Female | 3993 | | Left | 1.96 (2.74) | | 7.35 (4.90) |
| 19 | 45 | GBM | Female | 127084 | | Left | Data discarded due to technical issue | | |
| 20 | 28 | Astrocytoma | Male | 84788 | | Left | 1.02 (2.75) | | 3.15 (-0.41) |
| 21 | 45 | GBM | Female | 277109 | | Left | 2.83 (3.73) | | 5.47 (4.05) |
| 22 | 48 | Oligodendroglioma | Male | 153685 | | Left | 1.47 (1.85) | | 2.05 (1.60) |
| 23 | 42 | Astrocytoma | Male | 44270 | | Left | 4.29 | | -1.92 |
| 24 | 56 | Astrocytoma | Female | 78212 | | Right | 1.25 | | -0.61 |
| 25 | 71 | GBM | Male | 207337 | | Left | 0.67 | | 1.84 |
| 26 | 36 | GBM | Male | 84346 | | Right | 1.63 | | 4.63 |
| 27 | 37 | Cavernous malformation | Female | 183 | | Left | 1.01 | | 4.05 |
| 28 | 21 | Cavernous malformation | Female | 1708 | | Right | 0.52 (1.62) | | 5.81 (-0.73) |
| 29 | 63 | GBM | Male | 79084 | | Left | Data discarded due to technical issue | | |
| 30 | 68 | AVM | Female | 50224 | | Right | Could not localize the LMFG ROI | | |
| 31 | 61 | GBM | Female | 133252 | | Right | Could not localize the LMFG ROI | | |
| 32 | 43 | AVM | Female | 12456 | | Left | Data discarded due to technical issue | | |
| 33 | 66 | Astrocytoma | Male | 34734 | | Left | 2.26 | | 6.64 |
| 34 | 67 | Oligodendroglioma | Male | 42216 | | Left | 1.36 (1.35) | | 5.60 (3.96) |
| 35 | 67 | GBM | Male | 18604 | | Left | Could not localize the LMFG ROI | | |
| 36 | 28 | GBM | Male | 193209 | | Left | 2.33 (2.63) | | -0.25 (0.22) |
| 37 | 36 | Astrocytoma | Female | 64557 | | Left | 3.61 (5.46) | | 3.82 (2.76) |
| 38 | 49 | GBM | Female | 11801 | | Left | 3.70 (4.50) | | 3.79 (1.71) |
| 39 | 65 | Astrocytoma | Male | 29215 | | Right | 3.46 (4.83) | | -0.6 (-0.42) |
| 40 | 55 | Astrocytoma | Female | 15297 | | Left | 1.43 (2.34) | | 2.32 (1.62) |
| 41 | 20 | Astrocytoma | Male | 7570 | | Left | 2.20 (3.89) | | 5.87 (4.64) |
| 42 | 23 | Astrocytoma | Male | 19704 | | Left | 2.12 (2.95) | | 2.73 (1.92) |
| 43 | 57 | Oligodendroglioma | Male | 9308 | | Left | 1.16 (1.81) | | -0.37 (0.25) |
| 44 | 32 | Oligodendroglioma | Male | 19057 | | Left | 1.61 (2.40) | | 5.97 (3.19) |
| 45 | 29 | Astrocytoma | Female | 69361 | | Left | Data discarded due to technical issue | | |
| 46 | 39 | AVM | Male | 12433 | | Left | 0.76 (0.55) | | 2.97 (2.89) |
| 47 | 75 | Astrocytoma | Female | 25094 | | Right | 1.10 | | 2.13 |
| 48 | 75 | GBM | Female | 60841 | | Left | 0.82 (0.72) | | 1.61 (1.60) |
| 49 | 18 | Astrocytoma | Male | 5641 | | Right | 0.63 | | 0.67 |
| 50 | 56 | GBM | Male | 45310 | | Left | 1.11 (-0.25) | | -1.90 (-1.39) |
| 51 | 28 | Astrocytoma | Male | 62731 | | Left | 1.48 (1.23) | | 4.12 (1.85) |
| 52 | 70 | Astrocytoma | Male | 102302 | | Left | 1.01 (0.75) | | 1.51 (1.52) |
| 53 | 44 | Astrocytoma | Male | 15861 | | Right | 0.70 | | 4.20 |
| 54 | 66 | GBM | Female | 6054 | | Left | 1.96 | | -0.93 |
| 55 | 18 | Cortical dysplasia | Female | 21027 | | Left | 0.22 (0.39) | | 1.02 (0.17) |
| 56 | 70 | GBM | Female | 38524 | | Right | Could not localize the LMFG ROI | | |
| 57 | 67 | GBM | Male | 199912 | | Left | 0.97 (1.14) | | 1.62 (1.22) |
| 58 | 52 | GBM | Female | 28292 | | Left | 1.23 (1.08) | | 2.33 (1.40) |
| 59 | 34 | Astrocytoma | Female | 278688 | | Left | 2.63 (2.80) | | 3.78 (2.88) |
| 60 | 51 | Gliosis | Male | 15281 | | Left | 1.24 (2.04) | | 4.17 (1.74) |
| 61 | 60 | Oligodendroglioma | Female | 17316 | | Left | 0.75 (1.76) | | 3.06 (0.55) |
| 62 | 25 | Astrocytoma | Male | 74908 | | Left | 0.64 (1.41) | | 5.19 (3.78) |
| 63 | 31 | Oligodendroglioma | Male | 66304 | | Left | 0.96 (1.69) | | 3.86 (1.63) |
| 64 | 59 | Glioma | Male | 77713 | | Right | 2.33 (3.02) | | 1.60 (1.09) |
| 65 | 24 | Oligodendroglioma | Female | 8505 | | Left | 2.48 (3.72) | | 0.83 (0.26) |
| 66 | 59 | Astrocytoma | Female | 12096 | | Right | 1.62 (2.19) | | 5.76 (4.91) |
| 67 | 38 | AVM | Male | 19622 | | Left | 1.27 | | -0.55 |
| 68 | 59 | GBM | Male | 116479 | | Left | 3.04 (3.48) | | 2.33 (1.23) |
| 69 | 62 | Oligodendroglioma | Female | 25170 | | Left | 2.69 (3.14) | | 1.40 (1.39) |
| 70 | 45 | Astrocytoma | Male | 110326 | | Left | 1.38 (1.68) | | 1.80 (1.14) |
| 71 | 27 | Cavernous malformation | Male | 9046 | | Right | 0.29 | | 2.43 |
| 72 | 70 | Astrocytoma | Male | 19704 | | Left | 1.75 (2.15) | | 2.21 (2.35) |
| 73 | 47 | Astrocytoma | Female | 85962 | | Right | 1.50 (2.13) | | -0.14 (-0.40) |
| 74 | 48 | GBM | Male | 129643 | | Left | Could not localize the LMFG ROI | | |
| 75 | 21 | AVM | Female | 9459 | | Left | 0.93 | | 0.60 |
| 76 | 48 | Astrocytoma | Female | 90718 | | Left | 2.62 (2.53) | | 3.57 (1.29) |
| 77 | 26 | Astrocytoma | Male | 12604 | | Right | 1.66 (2.34) | | 3.46 (2.35) |
| 78 | 40 | AVM | Female | 7833 | | Left | 1.76 (2.01) | | 5.68 (2.73) |
| 79 | 36 | Oligodendroglioma | Female | 43092 | | Right | 2.00 (3.48) | | 4.08 (3.50) |
| 80 | 26 | Gliosis | Male | 17726 | | Left | 2.65 (3.70) | | 5.99 (4.24) |
| 81 | 28 | Cortical dysplasia | Female | 39850 | | Right | 2.72 (3.05) | | 1.05 (1.51) |
| 82 | 40 | AVM | Male | 31740 | | Right | Could not localize the LMFG ROI | | |
| 83 | 53 | Astrocytoma | Male | 48438 | | Right | 0.75 | | 2.79 |
| 84 | 35 | AVM | Male | 10130 | | Left | 1.23 (2.23) | | 2.76 (2.68) |
| 85 | 40 | Oligodendroglioma | Female | 12465 | | Left | 1.74 (2.57) | | 4.95 (1.06) |
| 86 | 55 | GBM | Female | 129994 | | Right | Could not localize the LMFG ROI | | |
| 87 | 42 | GBM | Male | 192798 | | Left | 0.76 | | 0.83 |
| 88 | 76 | GBM | Male | 1538 | | Left | Could not localize the LMFG ROI | | |
| 89 | 65 | GBM | Female | 102966 | | Right | 1.23 (1.24) | | -0.42 (0.33) |
| 90 | 28 | Oligodendroglioma | Male | 37022 | | Left | 3.13 (4.10) | | 0.33 (0.76) |
| 91 | 46 | DNET | Female | 9364 | | Left | 2.60 | | 5.74 |
| 92 | 79 | GBM | Female | 35746 | | Left | 0.67 | | -1.79 |
| 93 | 76 | GBM | Male | 41761 | | Left | 1.63 | | 5.10 |
| 94 | 65 | AVM | Male | 33915 | | Left | 1.58 (1.63) | | 2.40 (2.44) |
| 95 | 24 | Glioma | Male | 16251 | | Right | 1.55 (2.09) | | 1.89 (1.56) |
| 96 | 39 | Astrocytoma | Male | 133291 | | Right | 0.48 (0.40) | | 2.13 (1.79) |
| 97 | 41 | Glioma | Male | 33495 | | Left | 0.95 | | 2.81 |
| 98 | 63 | GBM | Male | 16919 | | Left | 0.94 (4.37) | | 1.33 (-1.47) |
| 99 | 53 | Oligodendroglioma | Female | 64556 | | Left | 0.66 | | 7.54 |
| 100 | 33 | AVM | Female | 15557 | | left | 1.19 | | 0.33 |
| 101 | 43 | Oligodendroglioma | Female | 23096 | | Left | 2.56 | | 1.61 |
| 102 | 42 | Astrocytoma | Male | 102665 | | Left | 0.69 (2.76) | | 2.98 (-0.27) |
| 103 | 35 | Astrocytoma | Male | 47252 | | Left | 1.21 | | 2.74 |
| 104 | 40 | Astrocytoma | Female | 26814 | | Left | 1.88 | | 1.30 |
| 105 | 62 | GBM | Male | 83984 | | Left | 0.55 | | 5.54 |
| 106 | 75 | GBM | Male | 96475 | | Left | Could not localize the LMFG ROI | | |
| 107 | 34 | Oligodendroglioma | Male | 71566 | | Left | 2.24 (3.06) | | 5.57 (4.05) |
| 108 | 22 | Astrocytoma | Male | 124642 | | Right | 0.96 (0.18) | | 0.14 (-1.18) |
| 109 | 44 | PLNTY tumor | Male | 14179 | | Left | 1.54 (2.16) | | -1.59 (-0.27) |
| 110 | 49 | Glioma | Female | 23800 | | Left | 1.30 | | 1.44 |
| 111 | 23 | Glioneuronal tumor | Male | 3871 | | Right | 1.65 (2.33) | | 1.95 (1.39) |
| 112 | 55 | GBM | Female | 85363 | | Right | 1.11 | | 3.08 |
| 113 | 23 | Astrocytoma | Male | 30201 | | Left | 2.25 (2.87) | | 0.35 (0.01) |
| 114 | 30 | Oligodendroglioma | Male | 220690 | | Left | Data discarded due to technical issue | | |
| **B.** Neurotypical participants | | | | | | | | | |
| Cumulative Participant ID | Age | Gender | Cross-validated T>AFP *t*-value | | Cross-validated P>AFT *t*-value | | |  | |
| 1 | 21 | Female | 0.36 | | 4.36 | | |  | |
| 2 | 19 | Male | 0.53 | | 3.28 | | |  |  |
| 3 | 22 | Male | 0.37 | | -2.13 | | |  |  |
| 4 | 22 | Male | 2.01 | | 1.57 | | |  |  |
| 5 | 20 | Male | 1.13 | | 0.32 | | |  |  |
| 6 | 19 | Male | 0.66 | | -0.41 | | |  |  |
| 7 | 18 | Male | 0.76 | | 2.24 | | |  |  |
| 8 | 24 | Female | 2.48 | | 2.43 | | |  |  |
| 9 | 26 | Male | 2.48 | | 1.62 | | |  |  |
| 10 | 20 | Female | 1.16 | | 1.53 | | |  |  |
| 11 | 20 | Female | 1.55 | | 5.17 | | |  |  |
| 12 | 19 | Female | 2.28 | | 0.64 | | |  |  |
| 13 | 21 | Female | 1.17 | | 1.29 | | |  |  |
| 14 | 19 | Female | 1.56 | | -0.47 | | |  |  |
| 15 | 23 | Male | 2.04 | | -1.04 | | |  |  |
| 16 | 20 | Female | 1.80 | | 3.46 | | |  |  |
| 17 | 21 | Female | 0.99 | | 0.08 | | |  |  |
| 18 | 19 | Female | 1.29 | | 3.09 | | |  |  |
| 19 | 20 | Male | 0.52 | | 4.92 | | |  |  |
| 20 | 22 | Female | 1.03 | | 5.11 | | |  |  |
| 21 | 19 | Female | 0.66 | | 1.68 | | |  |  |
| 22 | 19 | Female | 1.07 | | 2.19 | | |  |  |
| 23 | 21 | Female | 2.06 | | -0.14 | | |  |  |
| 24 | 21 | Female | 1.20 | | 4.84 | | |  |  |
| 25 | 21 | Male | 0.64 | | 6.07 | | |  |  |
| 26 | 19 | Male | 1.33 | | 4.94 | | |  |  |
| 27 | 19 | Female | 0.36 | | 4.19 | | |  |  |
| 28 | 20 | Female | 1.23 | | 2.33 | | |  |  |
| 29 | 20 | Male | Could not localize the LMFG ROI | | | | |  |  |
| 30 | 21 | Male | 0.43 | | 2.29 | | |  |  |
| 31 | 23 | Male | 1.94 | | 2.79 | | |  |  |
| 32 | 19 | Female | 1.37 | | 2.51 | | |  |  |
| 33 | 21 | Male | 1.73 | | -0.11 | | |  |  |
| 34 | 21 | Female | 1.57 | | 3.56 | | |  |  |
| 35 | 27 | Female | 1.42 | | 3.95 | | |  |  |
| 36 | 20 | Male | 2.53 | | -4.85 | | |  |  |
| 37 | 26 | Male | Could not localize the LMFG ROI | | | | |  |  |
| 38 | 57 | Female | 0.96 | | -0.26 | | |  |  |
| 39 | 43 | Female | 1.26 | | 2.99 | | |  |  |
| 40 | 20 | Male | Could not localize the LMFG ROI | | | | |  |  |
| 41 | 20 | Female | 2.20 | | 5.42 | | |  |  |
| 42 | 24 | Male | 0.67 | | 0.79 | | |  |  |
| 43 | 30 | Male | 0.58 | | 7.56 | | |  |  |
| 44 | 23 | Female | 1.27 | | 3.73 | | |  |  |
| 45 | 21 | Female | 0.94 | | 5.28 | | |  |  |
| 46 | 23 | Female | 1.04 | | -1.75 | | |  |  |
| 47 | 24 | Male | 0.83 | | 2.36 | | |  |  |
| 48 | 20 | Female | 0.93 | | 0.49 | | |  |  |
| 49 | 21 | Male | 1.55 | | 1.17 | | |  |  |
| 50 | 20 | Male | 0.56 | | 3.28 | | |  |  |
| 51 | 22 | Male | 0.93 | | 3.2 | | |  |  |
| 52 | 21 | Female | 0.48 | | 5.47 | | |  |  |
| 53 | 22 | Male | 1.53 | | 3.83 | | |  |  |
| 54 | 21 | Female | 1.53 | | -2.67 | | |  |  |
| 55 | 21 | Male | 1.75 | | 7.64 | | |  |  |

**Supplemental Table 2.** Peak left hemisphere voxels identified in the VLAM analysis of manipulable objects overlapping with parietal subregions derived from the Julich Brain atlas. The number of significant voxels in each region is provided, and the percentage of significant voxels in each atlas region is listed in parentheses.

| Region Name | Peak MNI Coordinate (XYZ) | | | Statistical Value | Number of significant voxels in each atlas ROI |
| --- | --- | --- | --- | --- | --- |
| Inferior Parietal Lobule, PFcm | -49 | -40 | 21 | -2.32 | 1556 (58.9%) |
| Parietal Operculum, Subregion 1 | -50 | -32 | 20 | -2.28 | 64 (2.04%) |
| Inferior Parietal Lobule, PGa | -49 | -56 | 28 | -2.28 | 63 (1.37%) |
| Inferior Parietal Lobule, PFm | -48 | -56 | 28 | -2.28 | 51 (0.90%) |
| Inferior Parietal Lobule, PF | -58 | -40 | 28 | -1.93 | 122 (2.92%) |
| Inferior Parietal Lobule, PFop | -59 | -28 | 18 | -1.79 | 63 (3.47%) |
| Anterior IPS, Subregion 1 | -36 | -46 | 32 | -1.68 | 1 (0.03%) |
| Inferior Parietal Lobule, PFt | -48 | -32 | 36 | -1.67 | 8 (0.17%) |

**Supplemental Table 3.** Voxelwise comparison between the VLAM analysis of manipulable objects (A) and places (B) in the left ventral OTC. The number of significant voxels in each region from the Harvard-Oxford cortical and subcortical parcellation atlases is provided, and the percentage of significant voxels in each atlas region is listed in parentheses (minimum threshold of 1%).

| Region Name | Region Number | Peak MNI Coordinate (XYZ) | | | Statistical Value | Number of Significant Voxels |
| --- | --- | --- | --- | --- | --- | --- |
| A. **Greater VLAM effect for manipulable object in left ventral OTC** | | | | | | |
| Posterior Supramarginal Gyrus | 39 | -56 | -44 | 32 | 13.30 | 214 (18.5%) |
| Parietal Operculum | 85 | -52 | -36 | 30 | 12.00 | 156 (26.6%) |
| Anterior Supramarginal Gyrus | 37 | -54 | -36 | 32 | 11.05 | 16 (1.7%) |
| Angular Gyrus | 41 | -52 | -50 | 18 | 9.65 | 93 (8.4%) |
| Planum Temporale | 91 | -54 | -38 | 20 | 7.90 | 78 (13.5%) |
| Middle Temporal Gyrus, temporal-parietal-occipital territory | 25 | -46 | -52 | 12 | 6.13 | 50 (5.5%) |
|  | | | | | | |
| B. **Greater VLAM effect for places in left ventral OTC** | | | | | | |
| Posterior Middle Temporal Gyrus | 23 | -50 | -38 | -2 | 11.80 | 325 (22.2%) |
| Planum Polare | 87 | -44 | -14 | -2 | 11.77 | 39 (10.3%) |
| Anterior Superior Temporal Gyrus | 17 | -52 | 0 | -10 | 10.84 | 77 (25.0%) |
| Anterior Middle Temporal Gyrus | 21 | -56 | -8 | -12 | 9.69 | 67 (13.8%) |
| Posterior Superior Temporal Gyrus | 19 | -62 | -18 | -2 | 8.41 | 8 (1.9%) |
| Heschl’s Gyrus | 89 | -48 | -14 | 0 | 8.10 | 17 (4.8%) |
| Planum Temporale | 91 | -38 | -30 | 14 | 7.86 | 26 (4.5%) |
| Hippocampus | 9 | -26 | -34 | -4 | 7.12 | 41 (5.7%) |
| Posterior Fusiform Gyrus | 75 | -42 | -20 | -20 | 5.10 | 21 (2.3%) |

**Supplemental Figure 1. Voxelwise lesion overlap among 95 participants.** (A) Projected on the cortical surface is lesion overlap among participants for whom we could localize voxels preferring manipulable objects in the left ventral OTC. Warmer colors indicate greater lesion overlap among participants. (B) The same map is projected in volume space (Z-coordinate provided). The areas of greatest lesion overlap include the left anterior temporal lobe, hippocampus, insula, pre- and post-central gyri, the superior frontal gyrus, the posterior superior temporal gyrus, and the supramarginal gyrus.

**Supplemental Figure 2. Overlap analyses using spherical ROIs in the anterior supramarginal gyrus (aSMG) and area PF using literature-defined coordinates. A.** The result of the Voxel-based Lesion-Activity Mapping (VLAM) analysis reported in Figure 2 is projected here (red). Each voxel is a data point. In that analysis of 95 participants, we showed that reduced fMRI responses for manipulable objects in left ventral OTC were associated with lesion presence in the left supramarginal gyrus and anterior intraparietal sulcus (among other areas). In this analysis, we overlaid areas aSMG and PF from Osiurak and colleagues and Orban and colleagues, respectively, to determine if overlapping voxels were identified. We find there is no overlap between the aSMG sphere (blue) and voxels identified in the VLAM analysis of manipulable objects, nor is there overlap between the area PF sphere (green) and voxels identified in the VLAM analysis of manipulable objects. The VLAM-identified regions likely involve white matter relevant to those functional regions. Supplemental Table 2 provides information about overlap between atlas-defined subregions within the left inferior parietal lobule and voxels identified in the VLAM analysis of manipulable objects (including the aSMG and anterior intraparietal sulcus).

**Supplemental Figure 3. Two analyses demonstrate the location of left anterior intraparietal sulcus (aIPS) voxels identified in the voxel-based lesion-activity mapping (VLAM) analysis using functional criteria. A.** The result of the VLAM analysis reported in Figure 2 is projected here (red). Each voxel is a data point. In that analysis of 95 participants, we showed that reduced fMRI responses for manipulable objects in left ventral OTC were associated with lesion presence in the left supramarginal gyrus and anterior intraparietal sulcus (among other areas). In this analysis, we find there is overlap between the voxels identified in the VLAM analysis of manipulable objects and voxels that exhibit differentially greater BOLD responses for manipulable objects in neurotypical participants (cool colors) in the left aIPS (axial Z slices 33-35). The map in cool colors is from an analysis of 55 neurotypical participants (see Materials and Methods for details). **B.** The voxels identified in the VLAM analysis of manipulable objects (red) that overlap with the anterior intraparietal sulcus (axial Z slices 33-35) are identified in a second overlap analysis, this time using a Neurosynth-derived probabilistic map of aIPS voxels (cool colors). The map in cool colors is the result of a meta-analysis identifying the voxels frequently reported in prior studies mentioning ‘anterior intraparietal’ in the article title or abstract.

**Supplemental Figure 4. Voxel-based lesion-activity mapping (VLAM) analysis of manipulable objects and places using an even/odd approach to independently define and test voxels in the left ventral OTC (occipitotemporal cortex) of 64 participants. A.** VLAM is a statistical technique that relates the strength of fMRI responses in a given ROI to the presence or absence of lesion damage in any given voxel of the brain. Voxelwise statistical significance was determined using a permutation analysis in which alignment of the independent variable (fMRI responses) and the dependent variable (lesion maps) were randomly shuffled across participants, repeating the full analysis 10,000 times. Voxelwise z-scores were computed for the true data in relation to the mean and standard deviation of voxel-by-voxel null distributions; the resulting z-score map was set to a threshold of z < -1.65 (*p* < .05, one-tailed) to determine chance-level likelihood of identifying a significant effect in each voxel. We found that reduced manipulable objects preferences in the left ventral OTC are associated with lesions in the left supramarginal gyrus and the left anterior intraparietal sulcus (red-yellow). Voxels in the lateral temporal lobe and white matter adjacent to the hippocampus were associated with reduced place preferences in the left ventral OTC. **B.** The VLAM result is projected on a volume-based rendering, with MNI Z coordinate provided above each axial image.

**Supplemental Figure 5. Replication of the voxel-based lesion-activity mapping (VLAM) analysis of manipulable objects after covarying lesion etiology in 95 participants.** A VLAM analysis of manipulable objects was conducted entering each subtype of lesion etiology as a covariate. This approach allowed us to systematically investigate the extent to which lesions to the left supramarginal gyrus and anterior intraparietal sulcus led to reduced functional neural responses in the left ventral OTC after accounting for lesion etiology. As can be seen in Panel N, we find that across the 13 etiologies (represented in Panels A through M) lesions to a common site that includes the left supramarginal gyrus, the left anterior intraparietal sulcus, and the left posterior temporal lobe are associated with reduced neural responses for manipulable objects in the left ventral OTC. The red-to-yellow scale represents the range of z-values for maps in Panels A through M. Voxels overlapping across the 13 maps are represented as a binarized map in Panel N.

**Supplemental Figure 6. Replication of the connectometry analysis of manipulable objects after covarying lesion etiology in 75 participants.** A connectometry analysis of manipulable objects was conducted entering each subtype of lesion etiology as a covariate. This approach allowed us to systematically investigate the extent to which reduced fractional isotropy of the left inferior fronto-occipital fasciculus, the left inferior longitudinal fasciculus, the left arcuate fasciculus, and the left middle longitudinal fasciculus was associated with reduced functional responses in the left ventral occipitotemporal cortex (OTC), after accounting for lesion etiology. As can be seen in Panel N, we find that across the 13 analyses (represented in Panels A through M) those 4 temporal lobe white matter fiber tracts are consistently associated with reduced neural responses for manipulable objects in the left ventral OTC. Overlaid upon these tracts is the result of the corresponding voxel-based lesion-activity mapping (VLAM) analysis entering the lesion etiology as a covariate. The results illustrate the excellent correspondence between VLAM and connectometry analyses of manipulable objects in left ventral OTC. Voxels overlapping across the 13 maps are represented as a binarized map in Panel N.

**Supplemental Figure 7. Comparison of voxel-based lesion-activity mapping** **(VLAM) results of manipulable objects (red) in 95 participants and voxels with adequate statistical power to detect a significant relation between functional neural responses and lesion damage.** As can be seen, the regions showing a significant VLAM effect for manipulable objects are a small subset of regions exhibiting sufficient statistical power to identify a significant effect.

**Supplemental Figure 8. Participants who produce spatial errors when gesturing object use have weaker preferences for manipulable objects in left ventral occipitotemporal cortex (OTC) relative to participants who do not produce spatial errors.** **A.** The left ventral OTC was defined using the whole-brain contrast of ‘Tools > [Animals, Faces, and Places]’ (equally weighted), in the group of 55 neurotypical participants (FDR-corrected; q < .05). This ROI was used to extract manipulable object preferences in the left ventral OTC using the contrast of ‘Tools > [Animals, Faces, and Places]’ (equally weighted) in 23 participants with brain lesions, resulting in the mean contrast-weighted t-value of all voxels in the independently defined ROI. **B.** The study began in 2015 and is ongoing. Prior to the pandemic, much of the focus of cognitive testing was on language comprehension and production, verbal working memory, and visual object recognition. Object use gesturing was not investigated. Because of the constraints on in-person testing during the COVID-19 pandemic, behavioral data acquisition was not possible between 2020-2022. We began in-person testing in earnest in 2023. It is for this reason that our analysis of fMRI responses and object use gesturing data is limited to data collected between 2023-2025.Twenty-three patient participants took part in a 25-trial object use gesturing task. On every trial, an image of a manipulable object was presented on a computer screen, and the participant was instructed to gesture object use with their dominant hand. Gesturing performance was video- and audio-recorded for offline analysis. A research assistant, blinded to the aims of this study, was trained to code the gestures. Inter-rater reliability was inspected and verified to be satisfactory (Cohen’s kappa ranged from 0.72 to 0.83). Gestures were coded along four dimensions, following the taxonomy used in previous studies (e.g., see S1-S3): Content (e.g., gesturing the use of a toothbrush as a hairbrush), Spatial (e.g., body-part-as-object error, incorrect configuration of the fingers, hand, wrist, or forearm, bicep, and shoulder), Temporal (e.g., exaggerating the range of movement or speed), and Other (e.g., forgetting how to produce the gesture or perseverating). Content errors were rare (2 trials, representing 0.003% of all trials). When a content error was identified, the gesture was not coded along the remaining dimensions. Other errors were also rare (1 trial, representing 0.0015% of all trials). The mean accuracy of the temporal component of gestures was 91% (standard deviation = 9%; minimum = 68%; maximum = 100%). We focused our analysis on spatial errors (mean = 89%; standard deviation = 16%; minimum = 40%; maximum = 100%).

Fourteen of the 23 participants produced spatial errors when gesturing object use, whereas nine did not. Inspection of manipulable object preferences in left ventral OTC revealed that participants who produced spatial errors when gesturing object use had weaker manipulable object preferences (yellow plot in Panel B) relative to participants who did not produce spatial errors (blue plot in Panel B). A two-step process was used to quantify the difference between these distributions. In the first step, a Mann–Whitney U test revealed that the median value of manipulable object preferences in participants who produced spatial errors was smaller than the median value of manipulable object preferences in participants who did not produce errors (z = -2.36, *p* < .05).

In the second step, we randomly assigned the participants’ manipulable object preferences to one of two groups and re-ran the Mann–Whitney U test 10,000 times, from which we derived a distribution of z-values due to chance. We observed that the Mann–Whitney U test associated with the real data was outside the null distribution of Mann–Whitney U test results (z = 2.44, *p* < .05). We found that in 80 of the 10,000 permutation analyses, we observed a Mann-Whitney U-test result that was equal to or greater than the effect we report. In other words, in the permutation analysis, 99.2% of the time we did not observe the effect we report in panel B. The effect size was quantified using the Hodges–Lehmann estimator of the median difference, with 95% bootstrap confidence intervals (effect size = 0.80; 95% lower CI = 0.20; 95% upper CI = 1.40). These findings demonstrate the robustness of the median difference between groups by showing that lower CI is well above 0.

We then re-ran this pipeline using total lesion volume in place of manipulable object preferences in the left ventral OTC. We found that participants who produced spatial errors in object-use gesturing had lesion volumes that were similar to those of participants who did not produce spatial errors in object-use gesturing (z = 0.53), and that this value was within the range of values due to chance when randomly assigning lesion volumes to one of two groups and testing for a differences between the scrambled and real data (z = 0.54).

**Supplemental References**

S1.	Cubelli, R., Marchetti, C., Boscolo, G., & Della Sala, S. (2000). Cognition in action: Testing a model of limb apraxia. *Brain and Cognition*, *44*, 144-165.

S2.	Garcea, F. E., Greene, C., Grafton, S. T., & Buxbaum, L. J. (2020). Structural disconnection of the tool use network after left hemisphere stroke predicts limb apraxia severity. *Cerebral Cortex Communications*, *1*, tgaa035.

S3.	Power, E., Code, C., Croot, K., Sheard, C., & Gonzalez Rothi, L. J. (2010). Florida Apraxia Battery–Extended and Revised Sydney (FABERS): Design, description, and a healthy control sample. *Journal of Clinical and Experimental Neuropsychology*, *32*, 1-18.
